# The burst of satellite DNA in *Leptidea* wood white butterflies and their putative role in karyotype evolution

**DOI:** 10.1093/dnares/dsae030

**Published:** 2024-10-26

**Authors:** Diogo Cavalcanti Cabral-de-Mello, Atsuo Yoshido, Diogo Milani, Jindra Šíchová, Ken Sahara, František Marec

**Affiliations:** Departamento de Biologia Geral e Aplicada, UNESP – Univ Estadual Paulista, Instituto de Biociências/IB, Rio Claro, São Paulo 13506-900, Brazil; Biology Centre of the Czech Academy of Sciences, Institute of Entomology, Branišovká 31, 370 05 České Budějovice, Czech Republic; Departamento de Biologia Geral e Aplicada, UNESP – Univ Estadual Paulista, Instituto de Biociências/IB, Rio Claro, São Paulo 13506-900, Brazil; Biology Centre of the Czech Academy of Sciences, Institute of Entomology, Branišovká 31, 370 05 České Budějovice, Czech Republic; Faculty of Agriculture, Iwate University, Morioka, Iwate 020-8550, Japan; Biology Centre of the Czech Academy of Sciences, Institute of Entomology, Branišovká 31, 370 05 České Budějovice, Czech Republic

**Keywords:** chromosome mapping, cryptic species, genome evolution, Lepidoptera, repetitive DNA

## Abstract

Satellite DNAs (satDNAs) are abundant components of eukaryotic genomes, playing pivotal roles in chromosomal organization, genome stability, and evolution. Here, we combined cytogenetic and genomic methods to characterize the satDNAs in the genomes of *Leptidea* butterflies. *Leptidea* is characterized by the presence of a high heterochromatin content, large genomes, and extensive chromosomal reshuffling as well as the occurrence of cryptic species. We show that, in contrast to other Lepidoptera, satDNAs constitute a considerable proportion of *Leptidea* genomes, ranging between 4.11% and 11.05%. This amplification of satDNAs, together with the hyperactivity of transposable elements, contributes to the substantial genome expansion in *Leptidea*. Using chromosomal mapping, we show that, particularly LepSat01-100 and LepSat03-167 satDNAs, are preferentially localized in heterochromatin exhibiting variable distribution that may have contributed to the highly diverse karyotypes within the genus. The satDNAs also exhibit W-chromosome accumulation, suggesting their involvement in sex chromosome evolution. Our results provide insights into the dynamics of satDNAs in Lepidoptera genomes and highlight their role in genome expansion and chromosomal organization, which could influence the speciation process. The high proportion of repetitive DNAs in the genomes of *Leptidea* underscores the complex evolutionary dynamics revealing the interplay between repetitive DNAs and genomic architecture in the genus.

## 1. Introduction

The majority of eukaryotic genomes consist of repetitive DNA sequences. These repeats are scattered as transposable elements (TEs) or organized in tandems, mainly as satellite DNAs (satDNAs) and some multigene families. Both classes of repetitive DNA are highly dynamic in terms of sequence and copy number. They are mainly responsible for the variability of genome size and play an important role in the organization and evolution of the genome.^1–4^ In addition, a growing body of data suggests a link between repetitive DNA sequences and major chromosomal rearrangements. This is evidenced by the detection of chromosomal rearrangements (eg deletions, breaks, translocations, duplications, inversions) that frequently occur in heterochromatin blocks composed largely of repetitive DNA, or even at the border of these regions with euchromatin.^5–10^ Since the chromosomal rearrangements could act as reproductive barriers,^11–14^ the repetitive DNAs could ultimately influence the speciation processes.

The satDNAs are noncoding repeats that are mainly arranged head-to-tail and occur in high copy numbers, especially in the heterochromatin of pericentromeric and subtelomeric chromosomal regions so that they can be observed at the cytological level. However, they can also be found in euchromatin. They ubiquitously colonize the genomes of eukaryotes and in some cases are very abundant, accounting for more than 50% of genomes.^4^ From an evolutionary perspective, satDNAs are subject to the phenomenon of concerted evolution, which leads to greater homogenization of repeats at the genomic level than between genomes.^15–17^ According to the library hypothesis, related species share a common set of satDNAs inherited from a common ancestor. In the course of evolution, certain variants could be amplified so that the major satDNAs occur in different species.^17^ Differentiation of satDNAs between sibling species could act as a postzygotic reproductive barrier, which is detrimental in hybrids and leads to reproductive isolation.^18–20^ Recently, it has been shown that in *Drosophila* hybrids, differences in satDNA composition between species lead to chromocentre disruption, micronuclei formation and tissue atrophy, which is a possible explanation for the reproductive isolation associated with satDNAs.^21^

Moths and butterflies, insects of the order Lepidoptera, are among the most diverse and species-rich groups of organisms, comprising around 160,000 species.^22^ In terms of cytogenetics, the species of this order have holocentric chromosomes without a localized centromere and mostly WZ/ZZ sex chromosomes with female heterogamety. In addition, the chromosomes of most Lepidoptera species are numerous, small, and uniform. In the course of evolution, the karyotypes of this group have been shaped by various chromosome rearrangements such as fusions, fissions, and inversions, resulting in highly diverse chromosome numbers and sex chromosome systems.^23–26^ As for the repetitive DNA fraction, only a few satDNA have been characterized in Lepidoptera species, which generally show low representativeness of their genomes and scattered chromosomal distribution.^27–31^

Wood-white butterflies of the genus *Leptidea* (Pieridae), *L. reali*, *L. juvernica,* and *L. sinapis*, form a triplet of cryptic species that can hardly be distinguished by morphological analyses. These species can be partially distinguished by comparing the genitalia but reliably only with the help of chromosomal studies and DNA sequence analyses including barcoding.^32,33^ An unexpected level of cryptic diversity and exceptional inter- and intraspecific variation in chromosome number was documented in these Western Palaearctic *Leptidea* species. This karyotypic variability was caused by fissions, fusions, and translocations.^32,34–37^ Including one Eastern Palaearctic species, *L. amurensis*, diploid chromosome numbers vary between 2n = 51 and 122 and each species has a unique system of multiple sex chromosomes, namely W_1–3_Z_1–4_ in females of *L. juvernica*, W_1–3_Z_1–3_ in *L.sinapsis*, W_1–4_Z_1–4_ in *L. reali*^34^ and W_1–3_Z_1–6_ in *L. amurensis*.^38^ At least in the 3 Western Palaearctic species, the multiple sex chromosomes evolved in a common ancestor in which they did not yet play a role in speciation, but later structural rearrangements of the multiple sex chromosomes may have contributed to reproductive isolation, eventually leading to speciation.^36^

In addition to variation in chromosome number and multiple sex chromosomes, *Leptidea* species are characterized by the presence of large and abundant heterochromatin blocks distributed throughout the genome, including the autosomes, which may contribute to the remarkable karyotype diversity in this genus.^34,38^ This is an exceptional case among the Lepidoptera, where heterochromatin is largely restricted to the W chromosome and the major rDNA clusters.^25,39–42^ Moreover, the genome sizes of *Leptidea* species are large compared to other lepidopterans, presumably as a consequence of the occurrence of recent hyperactive TEs, mainly LINEs, DNA elements, and some unclassified TEs.^43^

Due to the recent separation of the 3 Western Palaearctic *Leptidea* species (2.5 to 3.6 million years ago^43^) and their highly rearranged and variable karyotypes observed between species and populations, the genus *Leptidea* has attracted attention as a model for understanding karyotype evolution and the role of chromosome rearrangements in speciation. Considering the presence of large genomes and a large amount of heterochromatin with a high content of repetitive DNA,^34,38,43^ which could be related to the chromosomal variability of *Leptidea* species and, consequently, to the processes of speciation, our aim was to improve the understanding of the repetitive content in their genomes and its possible association with karyotype reshuffling. In this way, we identified the most abundant satDNAs populating the genomes of 3 *Leptidea* species (*L. juvernica*, *L. sinapsis,* and *L. reali*) through a computational analysis of next-generation sequencing data using RepeatExplorer/TAREAN.^44,45^ The analysis was combined with chromosome analyses in these 3 species and 2 Eastern Palaearctic species, *L. amurensis* and *L. morsei*, providing insights into satDNA and chromosome evolution in the genus. In addition, our cytogenetic mapping provided information on the composition and evolution of the W sex chromosomes.

## 2. Material and methods

### 2.1. *Genomic data and satellite DNA analysis*

For the genomic analysis, we selected reads from genomes that are available in Sequence Reads Achieve (SRA) and have already been published.^37,43^ A total of 17 sets of reads were used for satDNA characterization. These included 6 individuals of *L. juvernica* (3 from Ireland and 3 from Kazakhstan), 3 of *L. reali* from Spain, 6 of *L. sinapis* (3 from Sweden and 3 from Spain), one of *L. amurensis* from Mongolia, and one of *L. morsei* from China (**Table S1**).

The TAndem REpeat ANalyser (TAREAN)^45^ (https://repeatexplorer-elixir.cerit-sc.cz/) was used to identify satDNAs. A total of 9,000,000 paired-end reads were used as input to analyse the satDNAs within the Western Palaearctic species, which corresponds to 3,000,000 reads per individual, per species, per population. For *L. sinapis* from Spain, 664,771 reads were analysed with TAREAN, while 408,556 reads were analysed for individuals from Sweden. For *L. reali* from Spain, a total of 289,065 reads were analysed with TAREAN. In the case of *L. juvernica*, 264,542 reads and 236,832 reads were analysed for populations from Ireland and Kazakhstan, respectively. For *L. morsei* and *L. amurensis*, where only one genome per species was analysed, the TAREAN analysis was performed with input of 1,000,000 reads for each genome. For *L. morsei* 335,089 reads were analysed, while for *L. amurensis* 432,224 reads were analysed. All program options were selected according to default recommendations, and reads were preprocessed by quality filtering, pairing and formatting using the Galaxy platform’s ‘RepeatExplorer Utilities’.^44^ The putative satellites were then annotated and added to the *Leptidea* satDNA library. To exclude ambiguous satDNA sequences identified by TAREAN in all analysed *Leptidea* species, a homology test was performed by sequential sequence assembly and alignment comparison using Muscle^46^ implemented in Geneious v4.8 software.^47^ The nucleotide sequences of the identified satDNAs were deposited in GenBank under the accession numbers PQ298961-PQ298977.

RepeatMasker software version 4.1.1^48^ was used to estimate the genome abundance of satDNA families and Kimura’s 2-parameter (K2P) nucleotide divergence from each individual genome. The K2P divergence values are calculated based on the observed differences in nucleotide sequences, accounting for different substitution rates of transitions and transversions according to the K2P substitution model.^49^ Specific options were used, including ‘-no_is’ to skip bacterial insertions, ‘–lib’ to use a custom library, and ‘-s’ for a slow search that increases sensitivity by 0% to 5%. To calculate the K2P nucleotide divergence of each satDNA, we chose the ‘calcDivergenceFromAlign.pl’ script with the option ‘–a’ from the RepeatMasker utility tool,^48^ which generates results based on theK2P model. Prior to running RepeatMasker, given that certain groups of genomes were sequenced with different platforms and using reads of different sizes, we performed sequence preprocessing for each genome, which involved the use of the ‘rexp_prepare_normaltag.py’ script (available at https://github.com/fjruizruano/ngs-protocols). This script removed adapters and low-quality nucleotides (*Q* < 20) using Trimmomatic,^50^ resulting in approximately 0.9 to 1.0 Gb of data. For *L. morsei* and *L. amurensis*, whose sequenced genomes were exceptionally large (> 100 Gb of data), we randomly selected 2 × 3,000,000 reads from each genome using SeqTK (https://github.com/lh3/seqtk). For normalization, abundance values for a given satDNA family were calculated by summing all mapped nucleotides relative to the total number of nucleotides in the selected reads from each genomic library.

Abundance and divergence values for each satDNA were estimated separately for each individual and then averaged for each population (3 individuals each, except for *L. morsei* and *L. amurensis*, for which only one genome per species was used). Finally, the mean abundance and divergence of each population were averaged for each species and then for the *Leptidea* group comprising all 5 species analysed. The resulting data were used to rank the *Leptidea* satDNA collection in descending order of abundance. All data were also used for comparative analyses both between populations and between species.

To compare and understand the patterns of abundance variability among *Leptidea* species, specific statistical analyses were performed using either Student’s *t*-test or Wilcoxon matched-pairs test, depending on the results of the Shapiro-Wilk normality test. First, we compared the total satDNA abundance of *Leptidea* between species to test whether there were significant differences between groups, with the significance threshold set at *P* < 0.05. We then analysed the abundance of each satDNA in each species against each other to examine interspecific relationships, and each population against each other to assess intra-/interspecific relationships at the population level. Finally, for the 2 satDNAs with more than 1% of the genomes in the Western Palaearctic species (LepSat01-100 and LepSat03-167, see Results), we independently assessed statistical differences between populations by comparing their repeat frequencies. For all population tests, we only used species with triplicate genome samples (*L. juvernica*, *L. reali*, and *L. sinapsis*). All statistical analyses were performed using the Jamovi version 2.4.12 application (https://www.jamovi.org).

### 2.2. Insects and chromosome analysis

Adult specimens of *L. juvernica* and *L. sinapis* were collected in several localities in the Czech Republic. *Leptidea reali* was obtained from the Montseny area near Barcelona, Spain. The specimens were identified in previous studies by morphometric analysis of the genitalia and sequencing of the mitochondrial gene *COI* encoding cytochrome c oxidase subunit 1.^34^ These specimens originate from the same material studied earlier.^34,36^ Adult specimens of *L. amurensis* were collected near Mt. Takazasu, Yamanashi (Honshu Island), Japan,^38^ while specimens of *L. morsei* were collected in Hidaka (Hokkaido Island), Japan. The taxonomic identification of the 2 species, *L. amurensis* and *L. morsei*, was performed by sequencing a fragment of the *COI* gene, as previously described.^32^

For chromosomal mapping of satDNAs by fluorescence *in situ* hybridization (FISH), we used spread preparations of meiotic and mitotic chromosomes obtained from gonads (ovaries and testes) in previous studies.^34,36,38^ FISH mapping was performed for 2 abundant satellite DNAs, each representing more than 1% of the genome of at least one Western Palaearctic species. To prepare probes for FISH, monomers of each satellite DNA were amplified by polymerase chain reaction (PCR) in 2 consecutive reactions using the primer set LepSat01-100 (F: 5ʹ-AGGGTGCAGAGAACGATTTTG-3ʹ; R: 5ʹ-CGAGAACCTCGGATACGATAT-3ʹ) and LepSat03-167 (F: 5ʹ-ACACTTGAAACAGTAAGAGTTA-3ʹ; R: 5ʹ-TTGGAATGTTCACAACATGAC-3ʹ). Each reaction mixture consisted of 1 × Ex *Taq* PCR Rxn Buffer, 0.2 mM MgCl_2_, 0.16 mM dNTPs, 2 μM of each primer, 1 U TaKaRa Ex *Taq* DNA Polymerase (TaKaRa, Otsu, Japan), and 50–100 ng template DNA. The PCR conditions were as follows: initial denaturation at 94°C (5 min) and 30 cycles at 94°C (30 s), 57°C (30 s) and 72°C (80 s), with a final extension at 72°C for 5 min. For the first PCR, genomic DNA from each species served as template. The resulting PCR product was separated by 1.5% agarose gel electrophoresis. The band corresponding to the monomer of the respective satDNA was excised from the gel, purified using a Wizard SV Gel and PCR Clean-Up System (Promega, Madison, WI, USA) and used as a template for PCR re-amplification using the same primers and conditions. The purified monomer was used for probe generation by PCR labelling with either biotin-16-dUTP or digoxigenin-11-dUTP (both Jena Bioscience, Germany).

The FISH mapping procedure followed the previously described protocol.^51^ Probes labelled with biotin were detected with Cy3-conjugated streptavidin (Jackson Immuno Res. Labs. Inc., West Grove, PA, USA), while probes labelled with digoxigenin-11-dUTP were detected with sheep anti-digoxigenin-FITC conjugate (Sigma-Aldrich, St. Louis, MO, USA). Chromosomes were counterstained with DAPI (4ʹ,6-diamidino-2-phenylindole, dihydrochloride; Sigma-Aldrich) and slides were mounted with an antifade solution based on DABCO (1,4-diazabicyclo(2.2.2)-octane; Sigma-Aldrich).

To improve our understanding of the specific organization and to complement the information on the chromosomal organization of the satDNAs, we ran the CHRISMAPP script according to the parameters established previously^52^, using the chromosome-level genome assembly of *Leptidea sinapis* available in GenBank (accession number GCF_905404315.1). In this approach, Geneious 2023.2.1^53^ was used to annotate the consensus sequence of each satDNA family in the chromosome-scale genome assembly, based on a similarity threshold of 70%. The result was a.gff file with the annotations processed in Rstudio using the ggplot2 package.^54^ Finally, a bespoke script was implemented to visualize the annotations.

## 3. Results

### 3.1. *Unprecendent abundance and differential amplification of satellite DNAs in* Leptidea *genomes*

By applying the TAREAN protocol and RepeatMasker analysis in the 5 *Leptidea* species and their populations, a total of 17 satDNA families were identified, all of which were present in all species except one, LepSat17-88, which was absent in the *L. juvernica* genome library. These satDNAs were classified in decreasing order of abundance based on the mean of the 5 species. Among the different satDNA families, variations in the size of monomers were observed, ranging from 23 bp to 691 bp, with the mean A+T content of monomers ranging from 39.8% (LepSat17-88) to 73.5% (LepSat14-358), with a mean value of 59.9% (**Table 1**). Sequence homology analysis using GenBank and Repbase revealed no significant similarity with previously annotated repetitive elements. The abundance of satDNAs in the different genomes varied both for the total amount of satDNAs and for specific satDNAs, depending on the species and populations. The mean value of the total amount of satDNAs across all species was 8.01%. The total amount of satDNAs ranged from 4.11% in *L. sinapis* (approximately 26.4 Mb out of a genome size of 643 Mb) to 11.05% in *L. morsei*. In the other 3 species, the following intermediate values were observed: 6.85% (about 44.5 Mb from a genome size of 650 Mb) in *L. reali*, 10.53% (about 75.3 Mb from a genome size of 716 Mb) in *L. juvernica*, and about 7.5% in *L. amurensis* (**Table 1**). Furthermore, when comparing the 17 satDNAs between the 5 species and considering species pairs, significant statistical differences were only found between *L. reali* × *L. juvernica* and *L. reali* × *L. morsei* (**Table S2**).

**Table 1.** Characteristics of satDNAs identified in the genomes of *Leptidea* species, including monomer size in base pairs, average A+T content, abundance in the genome and K2P divergence. The genome size of the Western Palaearctic *Leptidea* species^43^ is given in brackets next to their names.

			*L. sinapsis* (643 Mb)		*L. reali* (650 Mb)		*L. juvernica* (716 Mb)		L. amurensis		L. morsei		Mean all	
SatDNAs	Monomer Size (bp)	A + T	K2P	Abundance	K2P	Abundance	K2P	Abundance	K2P	Abundance	K2P	Abundance	K2P	Abundance
LepSat01-100	100	57.0%	7.70%	2.98761%	6.93%	5.74805%	7.67%	7.49282%	10.27%	5.17428%	10.07%	7.58620%	8.53%	5.79779%
LepSat02-364	364	69.5%	9.34%	0.77011%	9.16%	0.72844%	9.32%	0.69783%	10.35%	0.00457%	9.85%	1.42352%	9.60%	0.72490%
LepSat03-167	167	67.7%	7.29%	0.10758%	7.10%	0.18475%	6.22%	1.52248%	9.03%	0.01772%	17.45%	1.25551%	9.42%	0.61761%
LepSat04-30	30	63.3%	9.75%	0.05673%	9.92%	0.04365%	10.08%	0.19587%	10.49%	1.48197%	10.26%	0.00452%	10.10%	0.35655%
LepSat05-557	557	65.9%	20.84%	0.02470%	20.67%	0.02429%	21.08%	0.02232%	2.14%	0.69489%	19.76%	0.15952%	16.90%	0.18514%
LepSat06-99	99	59.6%	11.57%	0.03688%	8.54%	0.03248%	13.72%	0.04376%	11.43%	0.08270%	8.29%	0.43348%	10.71%	0.12586%
LepSat07-28	28	60.7%	11.86%	0.04046%	11.97%	0.03589%	11.97%	0.40491%	11.25%	0.00229%	10.73%	0.00226%	11.55%	0.09716%
LepSat08-100	100	52.0%	5.46%	0.00998%	5.74%	0.00343%	4.90%	0.08269%	3.92%	0.00248%	5.82%	0.07265%	5.17%	0.03425%
LepSat09-23	23	60.9%	11.80%	0.04260%	10.99%	0.01997%	12.09%	0.03210%	9.85%	0.02482%	11.66%	0.03364%	11.28%	0.03062%
LepSat10-691	691	48.2%	0.91%	0.01173%	1.00%	0.01447%	1.23%	0.01287%	3.38%	0.00766%	1.79%	0.00607%	1.66%	0.01056%
LepSat11-93	93	60.2%	6.88%	0.00292%	6.76%	0.00131%	6.81%	0.00120%	8.39%	0.00096%	6.89%	0.03833%	7.15%	0.00894%
LepSat12-560	560	64.3%	9.52%	0.00920%	8.99%	0.00847%	8.99%	0.00789%	16.53%	0.00577%	11.67%	0.01164%	11.14%	0.00859%
LepSat13-203	203	57.1%	4.64%	0.00549%	5.38%	0.00284%	5.68%	0.00317%	6.83%	0.00563%	7.20%	0.00943%	5.95%	0.00531%
LepSat14-358	358	73.5%	5.79%	0.00339%	5.53%	0.00305%	4.56%	0.00266%	12.37%	0.00211%	5.86%	0.01424%	6.82%	0.00509%
LepSat15-141	141	61.7%	2.98%	0.00500%	3.10%	0.00580%	2.97%	0.00434%	7.19%	0.00121%	6.21%	0.00086%	4.49%	0.00344%
LepSat16-26	26	57.7%	5.55%	0.00064%	5.82%	0.00025%	5.25%	0.01286%	18.23%	0.00012%	17.92%	0.00011%	10.55%	0.00280%
LepSat17-88	88	39.8%	5.52%	0.00001%	5.10%	0.00001%	-	-	10.39%	0.00003%	0.29%	0.00325%	5.32%	0.00082%
Total abundance				4.11504%		6.85714%		10.53978%		7.50921%		11.05523%		8.01528%

The abundance of the most abundant satDNA, LepSat01-100, varied between species: it accounted for 7.49% of the genome in *L. juvernica*, 5.74% in *L. reali*, 2.98% in *L. sinapis*, and 5.17% and 7.58% in individuals of *L amurensis* and *L. morsei*, respectively. The mean abundance for the 5 species was 5.79%. In all cases, the other satDNAs had a much lower abundance compared to LepSat01-100 and mostly accounted for less than 1% of the genome. In *L. juvernica*, the lowest abundance was observed for LepSat11-93, which accounted for only 0.0012% of the genome, while LepSat17-88 had an abundance of 0.00001% in *L. reali* and *L. sinapis*. In *L. amurensis*, LepSat17-88 was also the least abundant with 0.00003% of the genome, while LepSat16-26 was only 0.00011% in *L. morsei* (**Table 1**).

**Fig. 1.**
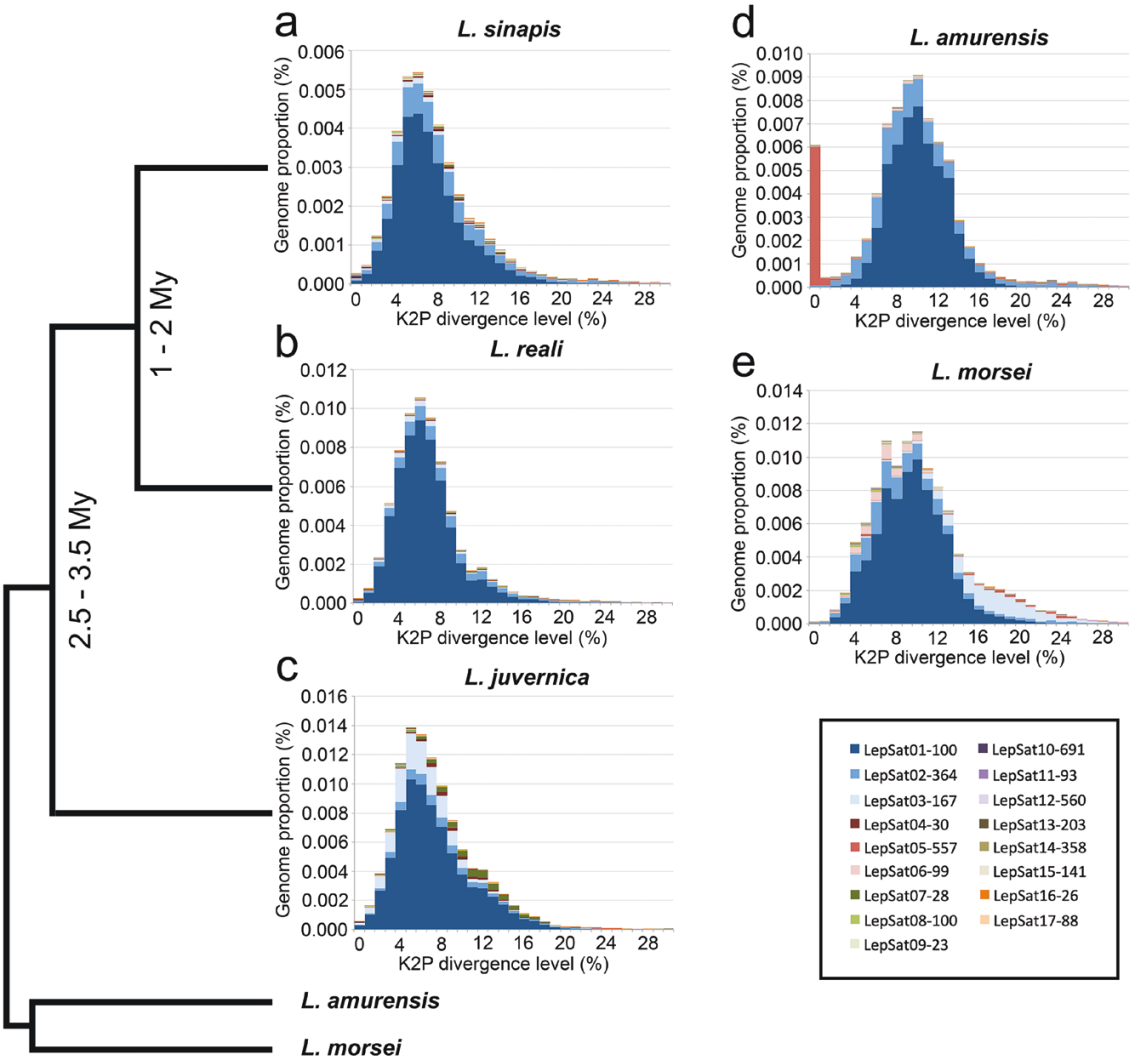
Repetitive satDNA landscapes (genome abundance *versus* K2P divergence), including the 17 identified satDNA families, of the 5 *Leptidea* species. (a) *L. sinapis*, (b) *L. reali*, (c) *L. juvernica*, (d) *L. amurensis*, and (e) *L. morsei*. Each satDNA family is represented by one colour. Note that for the 3 Western Palaearctic species, *L. sinapis*, *L. reali*, and *L. juvernica*, the peak of abundance tends to be on the left side compared to *L. amurensis* and *L. morsei*, indicating lower K2P divergence. The only exception is LepSat05-557 in *L. amurensis*. The time of divergence, if available, and the phylogenetic tree from previous studies^55^ (Talla et al. 2019) are shown on the left side of the panel.

**Fig. 2.**
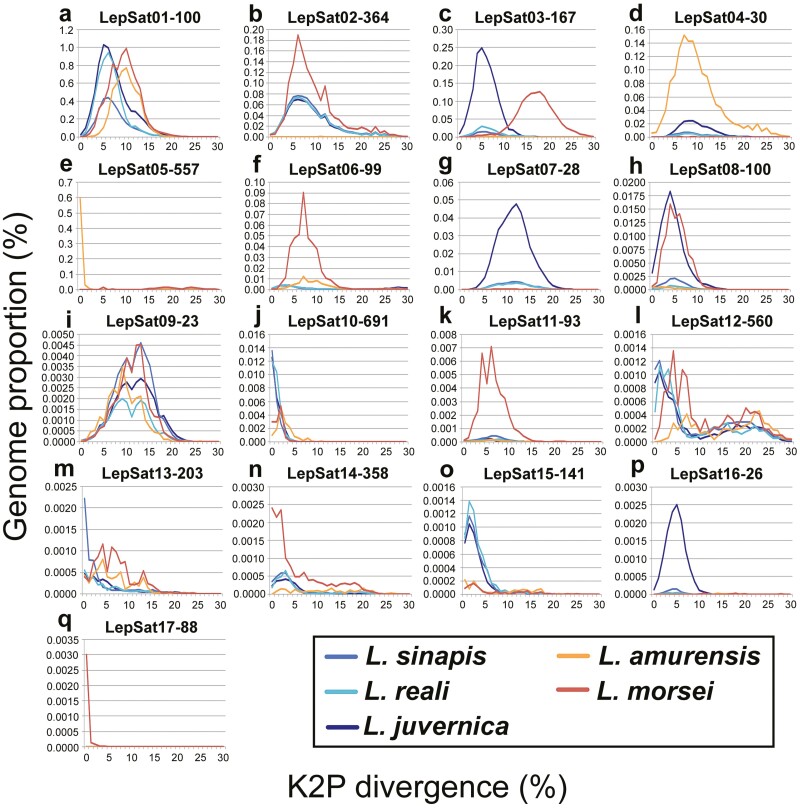
Comparative landscapes (genome abundance versus K2P divergence) for each of the 17 satDNAs in the 5 *Leptidea* species. Each species is represented by one colour as indicated directly in the image.

An analysis of satDNA landscapes (K2P divergence versus abundance) was performed to understand the patterns of amplification and homogenization of satDNAs. The mean divergence between satDNA families varied between species as follows: 8.08% in *L. sinapis*, 7.81% in *L. reali*, 8.28% in *L. juvernica*, 9.53% in *L. amurensis*, and 9.51% in *L. morsei* (mean value of K2P for each species in **Table 1**). The landscape analyses revealed an accumulation of satDNAs with slightly lower K2P divergence in the 3 Western Palaearctic species (**Fig. 1a–c**) compared to *L. amurensis* (**Fig. 1d**) and *L. morsei* (**Fig. 1e**). This pattern is particularly evident in LepSat01-100, where a peak in abundance was observed at K2P divergence values between 5 and 7 in the Western Palaearctic species, whereas in *L. amurensis* and *L. morsei*, the abundance peak was at a higher K2P divergence, between 9 and 11 (**Fig. 2a**). Abundance peaks at specific K2P divergences for all species were also observed in LepSat09-23 (**Fig. 2i**), which showed peaks around K2P of 9-14. In addition, different peaks were observed in the landscape for some satDNAs of different species. An obvious abundance peak with clear divergence was observed exclusively for one species each for LepSat02-364, LepSat06-99, LepSat11-93, LepSat14-358, and LepSat17-88 in *L. morsei* (**Fig. 2b, f, k, n, q**), LepSat04-30 and LepSat05-557 in *L. amurensis* (**Fig. 2d, e**), LepSat07-28 and LepSat16-26 in *L. juvernica* (**Fig. 2g, p**) and LepSat13-203 in *L. sinapis* (**Fig. 2m**). Abundance peaks for 2 species were found for LepSat03-167 and LepSat08-100 in *L. morsei* and *L. juvernica* (**Fig. 2c, h**), although the K2P values were different. While in LepSat08-100 the peak for the 2 species had similar K2P values, around 3–5, the peaks in LepSat03-167 were with different K2P values, around 4–6 for *L. juvernica* and a much less distinct peak for *L. morsei* with K2P values around 16–18. Two satDNAs had similar peaks for the 3 Western Palaearctic species, including LepSat10-691 with a K2P value of 0 and LepSat15-141 with a K2P value of 1 (**Fig. 2j, o**).

As we were interested in understanding the differences in satDNA abundance between the Western Palaearctic cryptic species, *L. juvernica*, *L. reali*, and *L. sinapis*, we performed statistical comparative analyses for the overall abundance of satDNAs between populations as well as analyses focussing on 2 satDNAs—LepSat01-100 and LepSat03-167—that had at least 1% abundance in one of the species. These satDNAs were selected because they are representative in their abundance within the species and may have greater significance for genomic organization. Analysis of all satDNAs revealed no statistical differences in abundance for almost all populations, except for the significant differences in satDNA abundance observed in *L. juvernica* (Ireland) × *L. reali* (Spain) and in *L. juvernica* (Ireland) × *L. sinapis* (Spain) (**Table S3**). The comparative analysis for LepSat01-100 showed similar abundance levels between populations, except between *L. juvernica* (Ireland) and the 2 populations of *L. sinapis* (Sweden and Spain) (**Table S4**). In contrast, the differences in abundance of LepSat03-167 were statistically significant in almost all comparisons, except between the 2 populations of *L. sinapis* studied from Spain and Sweden (**Table S5**).

### 3.2. *The heterochromatin of* Leptidea *chromosomes is enriched with satellite DNAs*

**Fig. 3.**
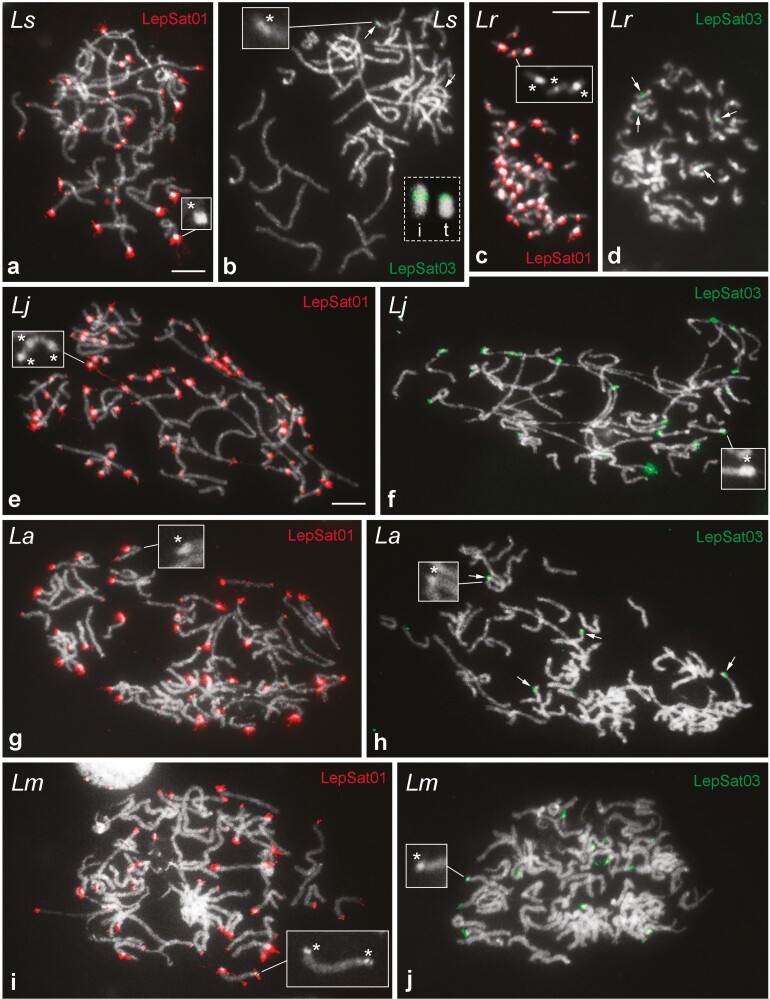
FISH mapping of LepSat01-100 (LepSat01) and LepSat03-167 (LepSat03) in male meiotic (a, b, e–j) and mitotic (c, d) chromosomes. (a, b) *L. sinapis*, *Ls*; (c, d) *L. reali*, *Lr*; (e, f) *L. juvernica*, *Lj*; (g, h) *L. amurensis*, *La*; (i, j) *L. morsei*, *Lm*. The insets (indicated by asterisks) in some panels highlight the heterochromatin blocks observed in the chromosomes of all species. Note in *L. sinapis* that the signals for LepSat03-167 are interstitial in one chromosome (i in inset of b) and terminal in another (t in inset of b), whereas in *L. reali* both signals are interstitial (d). In *L. juvernica* (f), *L. amurensis* (h) and *L. morsei* (j) the satDNA LepSat03-167 has multiple clusters. Bar = 10 µm.

We selected LepSat01-100 and LepSat03-167 for chromosomal mapping in all species because they occur with at least 1% abundance in the genome of at least one of the 3 Western Palaearctic species. PCR assays were used to determine a ladder pattern of monomers, dimers and multimers for both satDNAs, which is typically observed for true tandem DNAs such as satDNAs (**Fig. S1**). FISH mapping of LepSat01-100 and LepSat03-167 revealed a variable number of clusters in the male chromosomes of the 5 *Leptidea* species. In most cases, the clusters were located in the terminal region of the chromosomes, which is consistent with the distribution of the unusual heterochromatin blocks observed in these species. In most chromosomes, a satDNA cluster was located at only one terminus, but satDNA clusters were also detected in chromosomes with terminal heterochromatin blocks at both termini. The interstitial satDNA clusters also coincided with heterochromatic regions. In addition, small and scattered signals were also detected (**Fig. 3**). LepSat01-100 had a large number of clusters located in almost all chromosomes of the 5 species, alongside small, scattered signals that were more prominent in some species than others (**Fig. 3a, c, e, g, i**), while LepSat03-167 had a smaller number of clusters, the number and position of which varied depending on the species (**Fig. 3b, d, f, h, j**). In *L. sinapis* and *L. reali*, this satDNA was restricted to 2 pairs of chromosomes, with both signals occurring in an interstitial position in *L. reali* (**Fig. 3d**) and in *L. sinapis* (**Fig. 3b**), with one signal occurring in an interstitial position of one pair of chromosomes and the other signal occurring in a terminal position of another pair. In *L. juvernica*, *L. amurensis,* and *L. morsei*, multiple clusters were observed for LepSat03-167 (**Fig. 3f, h, j**).

**Fig. 4.**
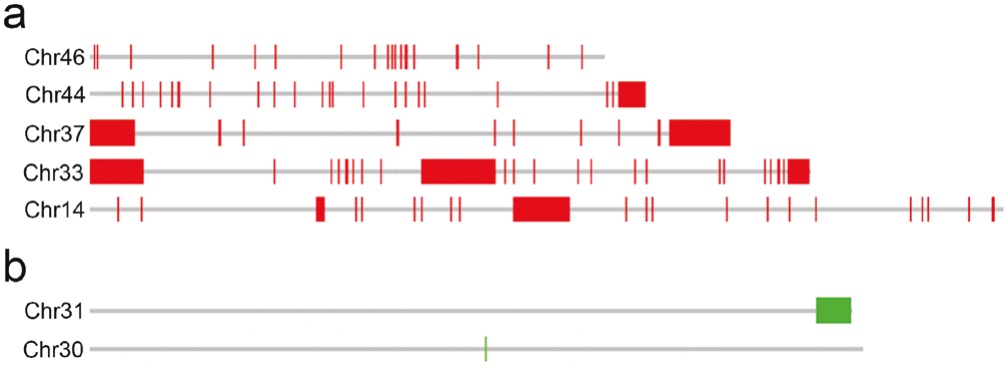
Examples of CHRISMAPP output for LepSat01-100 (a) and LepSat03-167 (b) on the chromosomes of *Leptidea sinapis*. (a) Chromosomes with no evident clusters (Chr 46), with one terminal cluster (Chr 44), 2 terminal clusters (Chr 37), 2 terminal, and one interstitial cluster (Chr 33) and with one interstitial cluster (Chr 14). (b) Chromosomes with an almost terminal cluster (Chr 31) and with one small signal (Chr 30).

CHRISMAPP analysis of satDNAs on the assembled chromosomes of *L. sinapis* provided a better resolution of the genome organization of the satDNAs and confirmed the chromosomal patterns for the 2 satDNAs mapped by FISH, ie LepSat01-100 and LepSat03-167. LepSat01-100 was observed on all chromosomes of *L. sinapis*, whereas LepSat03-167 was found only on a few chromosomes (**Fig. S2**). Different numbers of matches were observed for the different chromosomes, with arrays organized differently, including clustering in the distal region of the chromosome, an interstitial location and also without a clustering pattern (**Fig. 4**). In LepSat01-100, most chromosomes showed terminal or subterminal clustering, similar to FISH. Similarly, interstitial clustering was detected on some chromosomes, and only a few matches were identified on other chromosomes, with no obvious large cluster detected. Some chromosomes had clusters at only one terminus, but chromosomes with clusters at both termini were also observed, and clusters were also detected in both the terminal and interstitial regions of the same chromosome (**Fig. 4a**). LepSat03-167 was identified in only 7 chromosomes, and in most of them only a few copies were found. Only 2 of the chromosomes showed an obvious clustering pattern that was terminal/subterminal (**Fig. 4b**), similar to FISH mapping (**Fig. 3b**). Mapping of the other satDNAs by CHRISMAPP showed a highly dispersed distribution of LepSat02-364, clustering of LepSat04-30 in 2 chromosomes and only a few dispersed clusters for the remaining satDNAs (**Fig. S2**). LepSat05-557 and LepSat17-88 were not detected by CHRISMAPP.

**Fig. 5.**
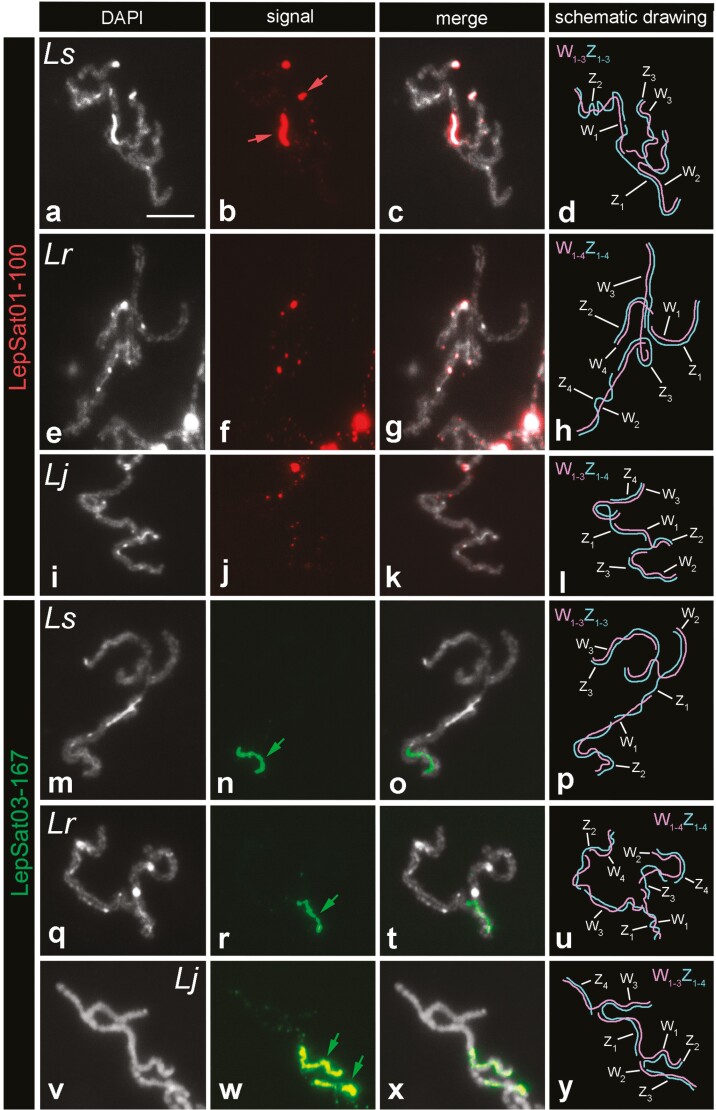
FISH mapping of LepSat01-100 and LepSat03-167 in selected sex chromosome multivalents from female pachytene nuclei of 3 Western Palaearctic *Leptidea* species: *L. sinapis* (a–d, m–p), *L. reali* (e–h, q–u), and *L. juvernica* (i–l, v–y). (a, e, i, m, q, v) DAPI, (b, f, j, n, r, w) probe signals, (c, g, k, o, t, x) merged DAPI plus signals, (d, h, l, p, u, y) schematic drawing of the sex chromosome multivalents (the individual sex chromosomes in the multivalents were identified based on the distribution of heterochromatin blocks and the specific mode of meiotic pairing between the W_n_ and Z_n_ chromosomes, as shown in previously published studies^34,36^). In the column with the probe signals, coloured arrows indicate strong and large signals. *Ls* (*L. sinapis*), *Lr* (*L. reali*), and *Lj* (*L. juvernica*). Bar = 10 µm.

Our analysis of meiotic pachytene complements in females of the Western Palaearctic species (*L. sinapis*, *L. reali,* and *L. juvernica*) revealed the presence of satDNAs on the sex chromosomes, mainly on the W chromosomes. In *L. sinapis*, LepSat01-100 covered the DAPI-positive W chromosome segments, including a large interstitial segment of the W_1_ and one end of the W_3_ chromosomes. Signals for this satDNA were also observed at the ends of the Z_3_ chromosome (**Fig. 5a–d**). In *L. reali* (**Fig. 5e–h**) and *L. juvernica* (**Fig. 5i–l**), only small dot signals were observed on the W and Z chromosomes. LepSat03-167 showed more intense signals covering large segments of the W chromosomes in all 3 species, including W_1_ in *L. sinapis* (**Fig. 5m–p**) and *L. reali* (**Fig. 5q–u**), and W_1_ and W_2_ of *L. juvernica* (**Fig. 5v–y**).

## 4. Discussion

In this study, the use of a combination of cytogenetic and genomic methods has enabled a comprehensive characterization of the predominant satDNA sequences in the genomes of *Leptidea* butterflies. This approach has provided detailed insights into the possible role of these satDNAs in genomic and chromosomal organization and evolution. In addition, we have analysed the dynamics of these satDNAs both before and after species divergence, contributing to a deeper understanding of evolutionary processes within the genus *Leptidea*. The importance of satDNAs for the genome organization of *Leptidea* species is reflected in the high abundance of this sequence type in the 5 species studied here compared to other Lepidoptera species in which the satellitome has already been investigated. Based on data collected using the same methods as those used here, satDNAs were found to account for only a small part of the genome in most Lepidoptera species, such as 0.215% of the genome of *Diatraea saccharalis*,^56^ 0.65% of the genome of *Spodoptera frugiperda*^31^ and about 0.14% of the genome of *Cydalima perspectalis*,^30^ while the proportion of satDNAs in *Leptidea* species ranges from 4.11% to 11.05%. However, to obtain a more accurate scenario about satellitomes of Lepidoptera, it is necessary to analyse other species with large genomes, such as those of *Leptidea* butterflies.

Compared to other moths and butterflies, *Leptidea* species generally have large genomes. According to a published study^43^, this increase in genome size is a consequence of the recent hyperactivity of TEs, which led to the accumulation of some unclassified TEs in *Leptidea* species after the split-off of other members of the Pieridae family. Moreover, our data clearly show that the rapid increase in genome size in *Leptidea* species was also driven by the amplification of satDNAs, as this type of sequence constitutes a significant part of their genomes, in contrast to other Lepidoptera.^30,31,56^ The causes and consequences of genome size variation from an evolutionary perspective are still debated, and in the absence of evidence for selection, neutral evolution is assumed.^57^ In the case of *Leptidea* species, the limited power of natural selection to prevent the spread of TEs outside functional genes explains their high abundance in the genome.^43^ Similarly, this could also apply to satDNAs that have spread in the genome of *Leptidea* species by passive amplification via various mechanisms such as DNA polymerase slippage. At least the massively amplified satDNA (LepSat01-100) is anchored in chromosomal regions where a low number of genes is expected, namely in the heterochromatic regions. Occasional amplification of satDNAs has also been reported in other insect groups, without evidence of adaptive traits, such as in *Melipona* bees,^58^ the blood-sucking hemipteran *Triatoma delpontei*^59^ and the beetle *Tenebrio molitor*.^60^

Although there is no direct evidence for the effect of satDNAs on the adaptive process, this type of sequences could serve as a source of chromosomal rearrangements and play a role in genomic differentiation, ultimately leading to the emergence of reproductive barriers.^4,19,61^ As previous studies have shown, chromosome rearrangements, especially fusions and fissions, occurred extremely frequently in *Leptidea* species during evolution.^34,37,38^ In this context, TEs and satDNAs may have played a role in this extreme genome reshuffling. We have shown that LepSat01-100 is mainly associated with the terminal regions of multiple chromosomes and in some cases forms large interstitial blocks (**Fig. 3**). These large blocks of satDNAs, together with the extensive heterochromatic blocks, are unusual for chromosomes of Lepidoptera. Normally, lepidopteran chromosomes have scattered satDNAs or only a few blocks are observed.^27,30,56^ We hypothesize that these large satDNA clusters result from the process of heterochromatin amplification and that they may play a role in chromosomal rearrangements. In this context, it is interesting to note that the highest number of interstitial satDNA clusters was observed in *L. reali*, the species with the lowest number of scaffolds (*n* = 26), suggesting that these satDNAs could possibly modulate fusion events. Furthermore, the enrichment of LepSat01-100 at the chromosome termini and in the interstitial regions was also confirmed in the assembled chromosomes of *L. sinapis* (**Fig. S2**).

For the other satDNA mapped by FISH on chromosomes, ie LepSat03-167, extensive differentiation was observed between species in terms of the number of clusters. In *L. juvernica*, *L. morsei* and to a lesser extent in *L. amurensis*, it was distributed in the form of large clusters at the end of the chromosomes, suggesting a significant involvement of this satDNA in chromosome differentiation between these species. However, in *L. sinapis* and *L. reali*, only 2 pairs of chromosomes were labelled for LepSat03-167, but these clusters were positioned differently, either terminally or interstitially (**Fig. 3**), suggesting the occurrence of chromosomal inversions between these species. Alternatively, differential amplification at different positions—whether terminal or interstitial—of previous small satDNA loci would also be possible.

According to the library hypothesis, the satDNAs of related species reflect their common ancestor and therefore the satellitomes of related species have similar satDNA compositions, with most variation being quantitative.^17,62^ Although the total amount of satDNAs was similar among the *Leptidea* species studied and significant statistical differences were only observed between *L. reali* and *L. juvernica* or *L. morsei*, the specific satDNA values differed between them, reflecting the rapid quantitative changes intrinsic to satDNAs. The overall quantitative changes in the satellitomes of *Leptidea* species reflect their phylogenetic history, as we observed a decrease in satDNA abundance in the 2 most closely related species, *L. sinapis* and *L. reali*. Another specific phylogenetic trend was the lower K2P divergence of repeats in the 3 cryptic species compared to *L. amurensis* and *L. morsei*, suggesting that homogenization of satDNAs occurred after the separation of this clade, or alternatively a higher accumulation of mutations in these 2 sister species. The quantitative changes in specific satDNA families were also accompanied by an increase or decrease in the number of chromosomal clusters. This can be observed in LepSat03-167, where the highest abundance was observed in *L. juvernica* and *L. morsei* with a high number of chromosomal clusters, while *L. amurensis* has a lower abundance and a smaller number of chromosomal clusters. In *L. sinapis* and *L. reali*, only 2 pairs of chromosomes harbour clusters and the abundance is very low. This concordance between the evolutionary patterns of satDNAs and the phylogenetic data suggests that the evolutionary histories of satDNAs and species are intertwined. Moreover, the differences in the profile of satDNAs and in the chromosomal distribution of these repeats that occurred during the separation of species could act as reproductive barriers, as previously hypothesized^18–20^ and contribute to the isolation of cryptic species that may be sympatric in some regions.

Our analysis of satDNA abundance in populations of Western Palaearctic species provides a more detailed scenario for understanding the involvement of satDNA in the process of species differentiation. The overall evolution of the satDNA library showed no differentiation at the population level within intraspecies status. However, significant differentiation was observed between the *L. juvernica* population from Ireland compared to the *L. reali* and *L. sinapis* populations from Spain. These data suggest that differentiation in the satDNA library is most effective only after the process of speciation. Furthermore, this differentiation was more pronounced in specific satDNAs, such as LepSatDNA01-100, which showed significant differentiation between *L. juvernica* (Ireland) and *L. sinapis* (Sweden and Spain). In other butterflies, the impact of repetitive DNAs at micro- and macro-evolutionary levels has recently been observed in the genus *Erebia*, where increased differentiation in repeat landscapes corresponds to overall genetic differentiation and where repeats appear to be associated with large chromosomal rearrangements that may lead to species diversification.^63^

The sex chromosomes of Western Palaearctic *Leptidea* species evolved from a common ancestor through repeated translocations, fusions, and fissions between ancestral sex chromosomes and autosomes. In addition, species-specific rearrangements likely contributed to reproductive isolation.^36^ Our data from mapping LepSat01-100 and LepSat03-167 in the female sex chromosomes of the 3 Western Palaearctic *Leptidea* species indicate that these chromosomes underwent species-specific patterns of satDNA amplification in addition to species-specific rearrangements. This resulted in dissimilarities in the W chromosomes at both intragenomic and intergenomic levels, which could contribute to the speciation process and accelerate the differentiation of these elements from their Z chromosome partners. The accumulation of satDNA repeats on the heteromorphic sex chromosomes is a common phenomenon observed in various animal groups,^64–66^ and it appears to be a fundamental feature of sex chromosome evolution. However, the accumulation of satDNAs on the sex chromosomes of *Leptidea* species differs from other lepidopteran species, as they occupy a large part of the W chromosomes and form large blocks, while in other species only localized clusters or scattered signals have been observed.^28,30,56^ This confirms the notion of a strong amplification of satDNAs in *Leptidea* species and apparently reflects their evolutionary history when considering the timescale, as the older W chromosomes, such as W_1_, were enriched with satDNAs, especially LepSat03-167.

In summary, our data reveal highly differentiated patterns of satDNA organization in *Leptidea* species compared to other Lepidoptera species studied so far, primarily caused by amplification during their evolutionary history, which has clearly contributed to the genome expansion of the genus. The satDNAs represent the major components of the heterochromatic blocks in these species and may have contributed to the process of chromosomal differentiation within the genus, possibly influencing the speciation process. Moreover, this class of genomic elements clearly played a role in sex chromosome differentiation. These data add a new layer of information to our understanding of the satellitome in the Lepidoptera, their diverse sex chromosome composition and evolution, and their putative role in the speciation of cryptic species. Genome analyses have established a link between specific TEs and structural chromosome rearrangements in *Leptidea* species, with a significant enrichment of LINEs and LTRs on chromosomes where fusions occurred.^37^ These data in conjunction with our analysis for satDNAs reveal an unprecedentedly complex evolutionary scenario of chromosome rearrangements in *Leptidea* species in which amplified repetitive DNAs may have been involved. Finally, our data provide insights into the impact of repeats on chromosomal rearrangements and speciation in species with holocentric chromosomes that have been poorly understood.

## Supplementary Material

dsae030_suppl_Supplementary_Figure_S1

dsae030_suppl_Supplementary_Figure_S2

dsae030_suppl_Supplementary_Table_S1

dsae030_suppl_Supplementary_Table_S2

dsae030_suppl_Supplementary_Table_S3

dsae030_suppl_Supplementary_Table_S4

dsae030_suppl_Supplementary_Table_S5

dsae030_suppl_Supplementary_Material

## Data Availability

Raw sequence reads are deposited in the SRA (ERX2099072, ERX2099073, ERX2099074, ERX2098946, ERX2098947, ERX2098948, ERX2098970, ERX2098971, ERX2098972, ERX2099075, ERX2099076, ERX2099077, ERX2099046, ERX2099047, ERX2099048, ERX11508160, ERX11508154).
