# Supplementary figures and images for: The burst of satellite DNA in Leptidea wood white butterflies and their putative role in karyotype evolution
Source: DNA Res. 2024 Oct 26;31(6):dsae030. doi: 10.1093/dnares/dsae030 (PMC11565590; doi:10.1093/dnares/dsae030)

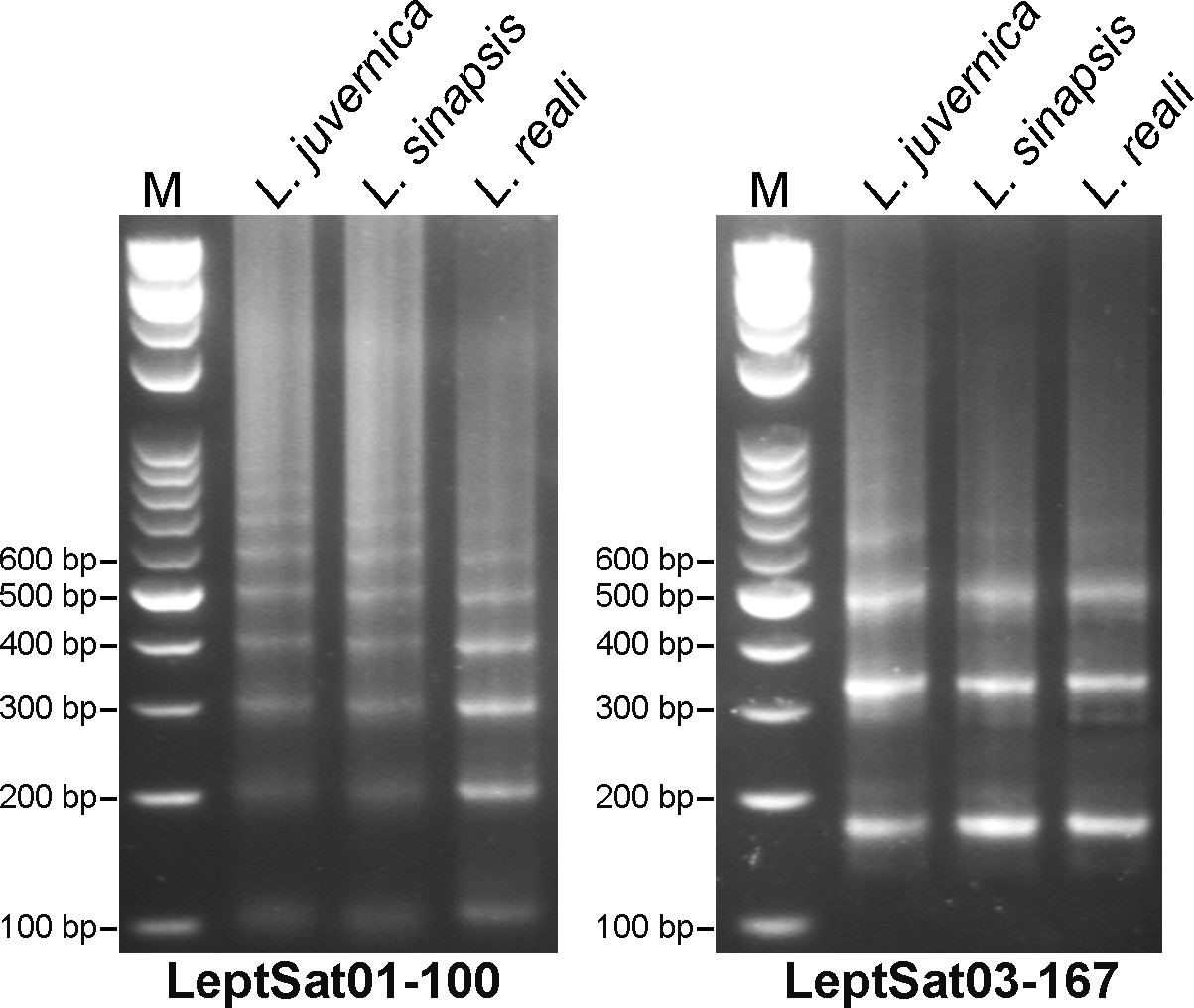

Supplement: dsae030_suppl_Supplementary_Figure_S1 [file dsae030_suppl_supplementary_figure_s1.jpeg]

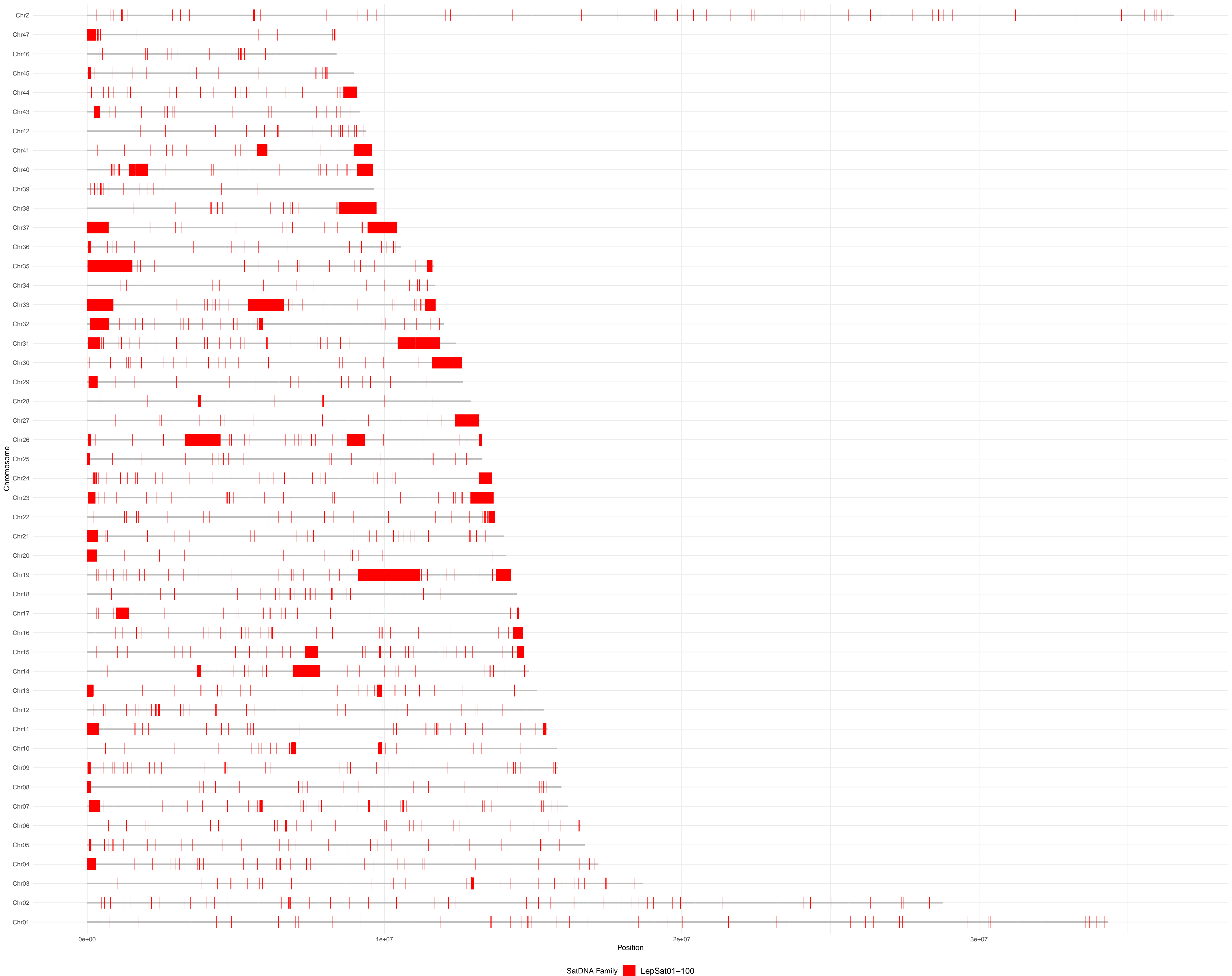

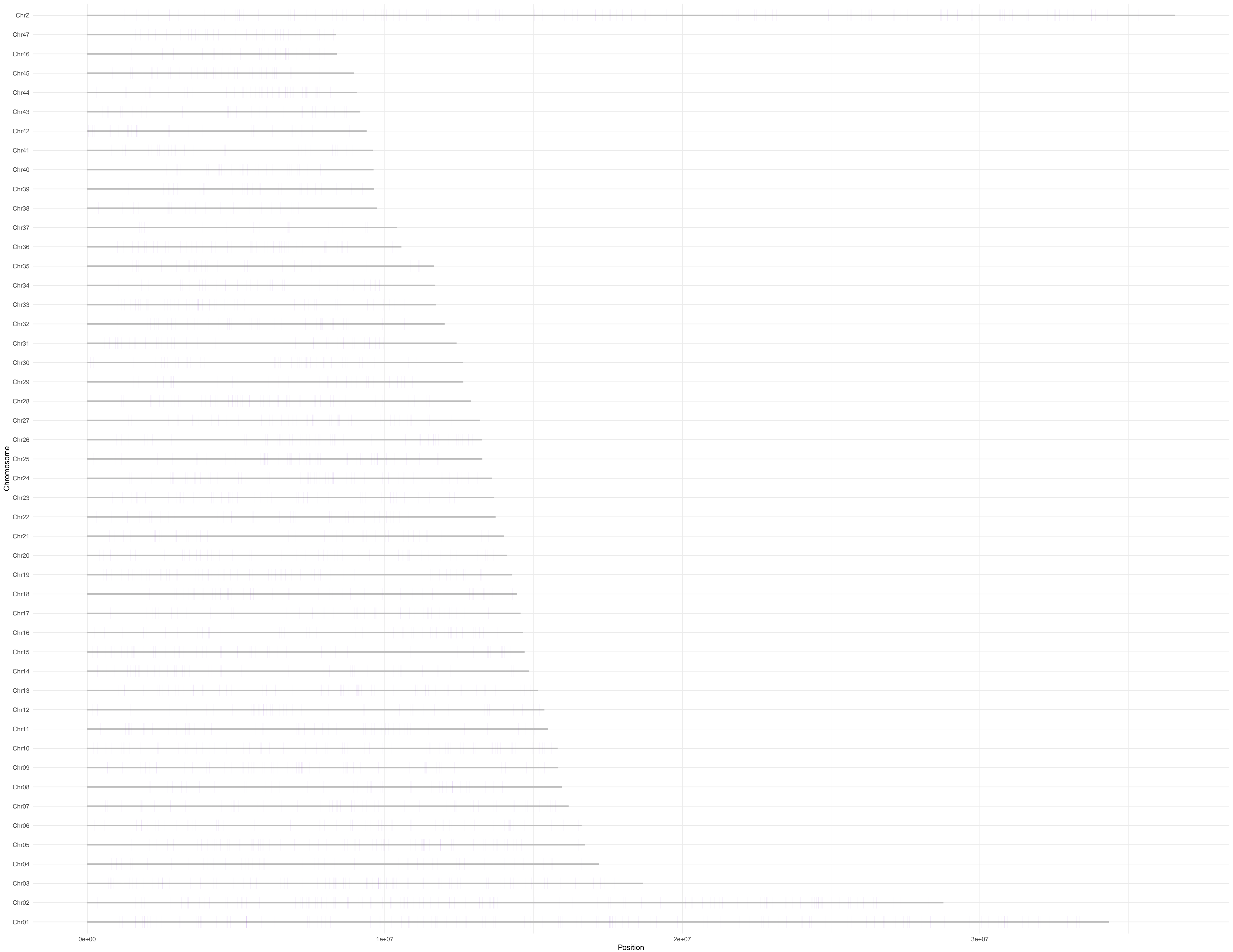

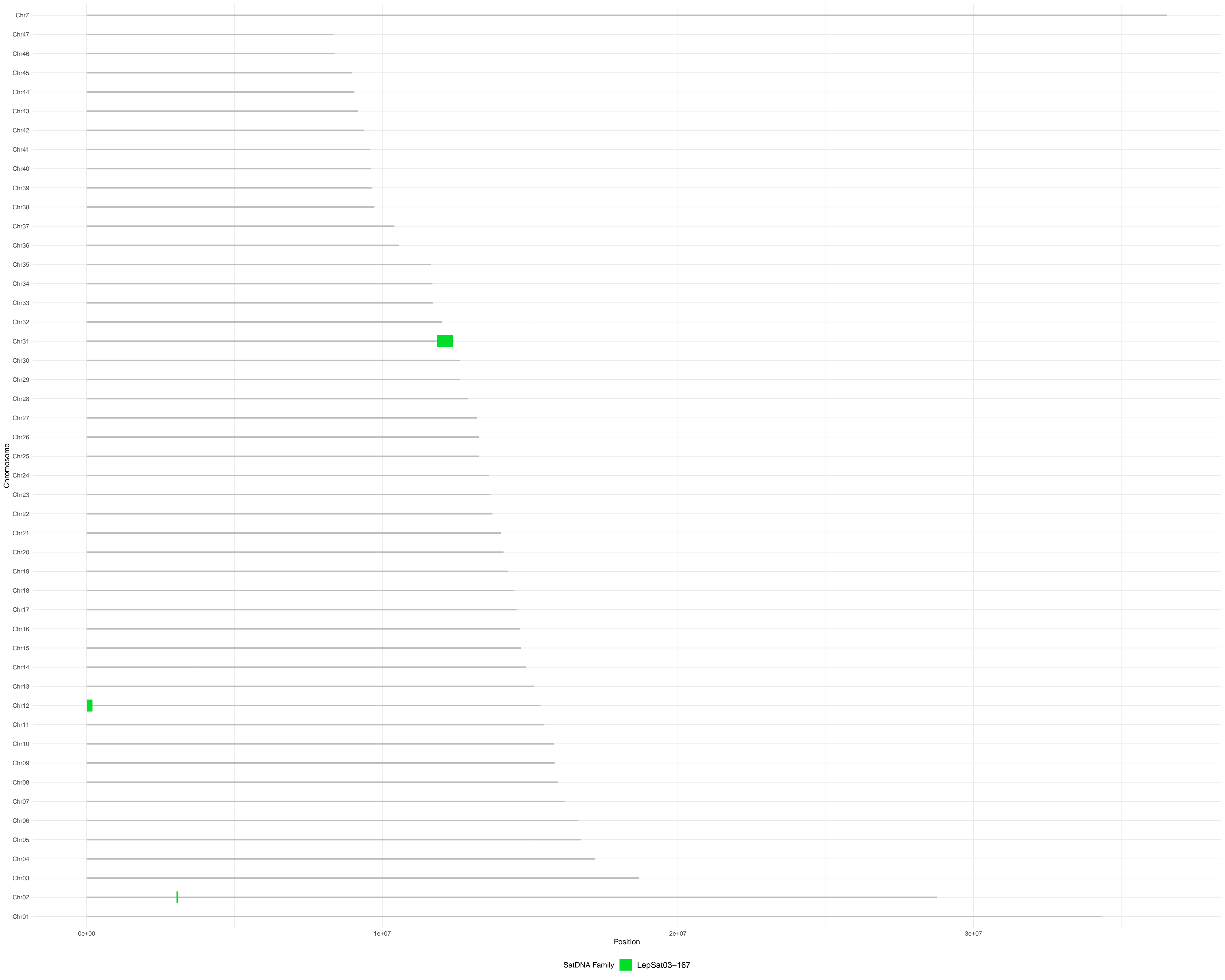

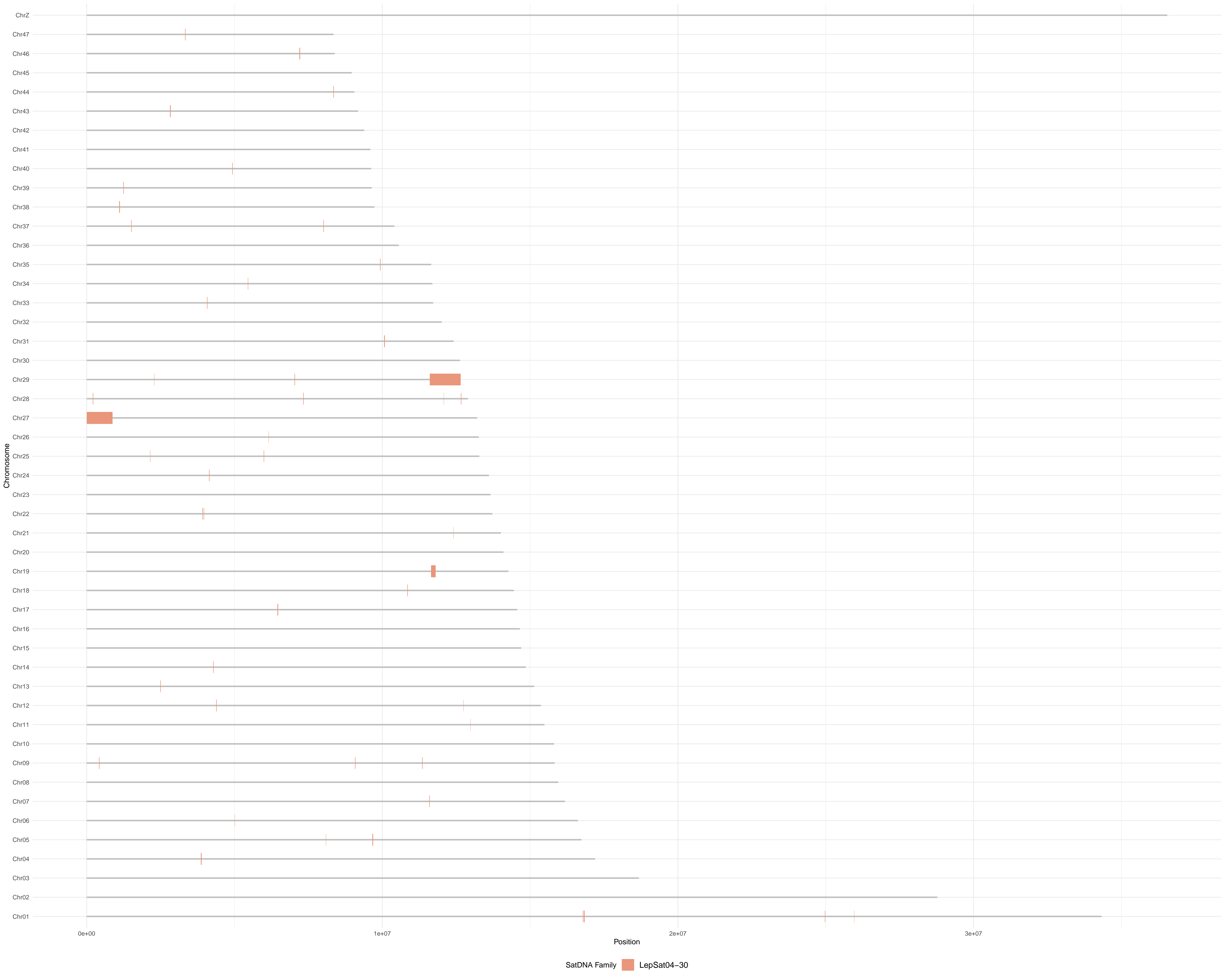

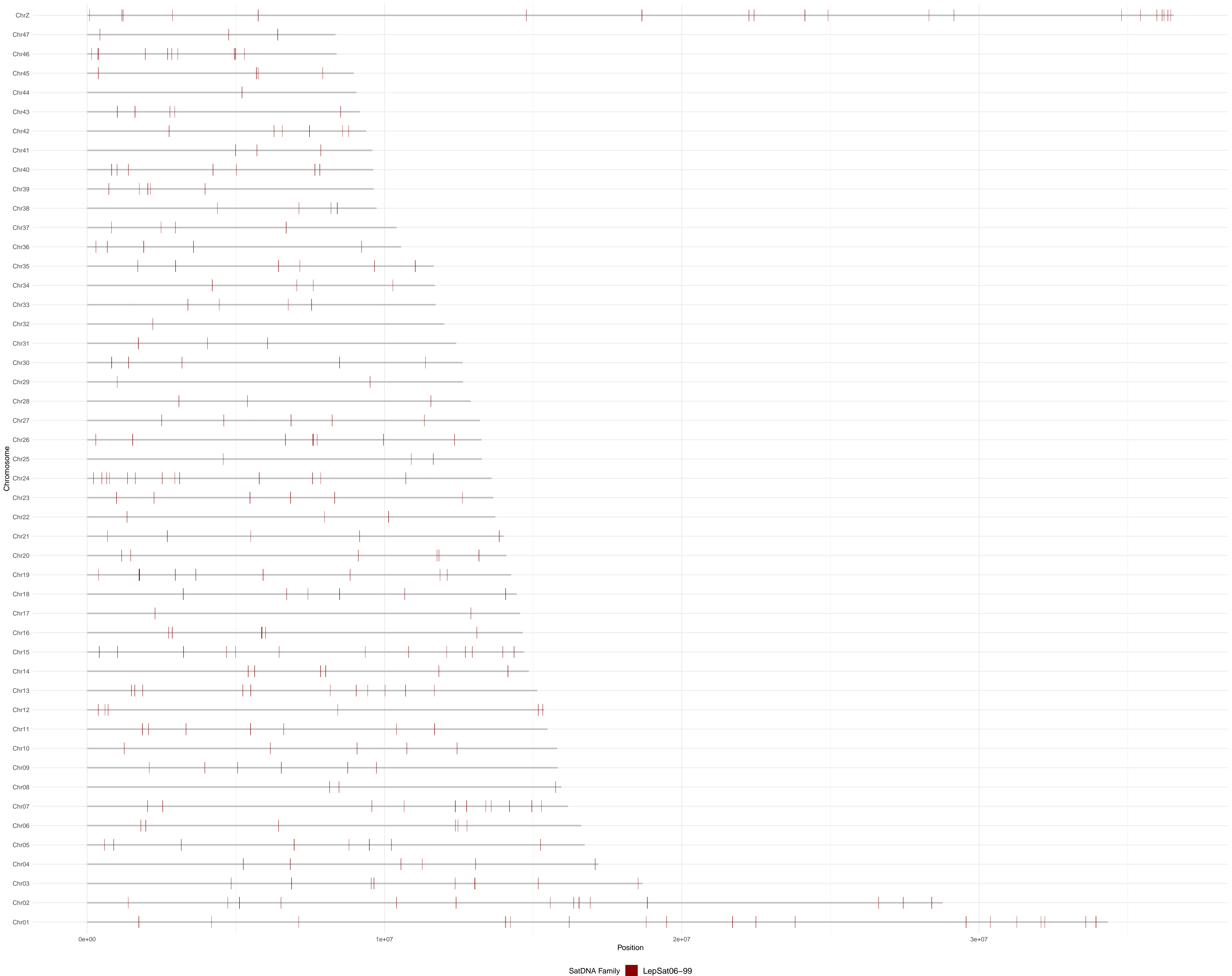

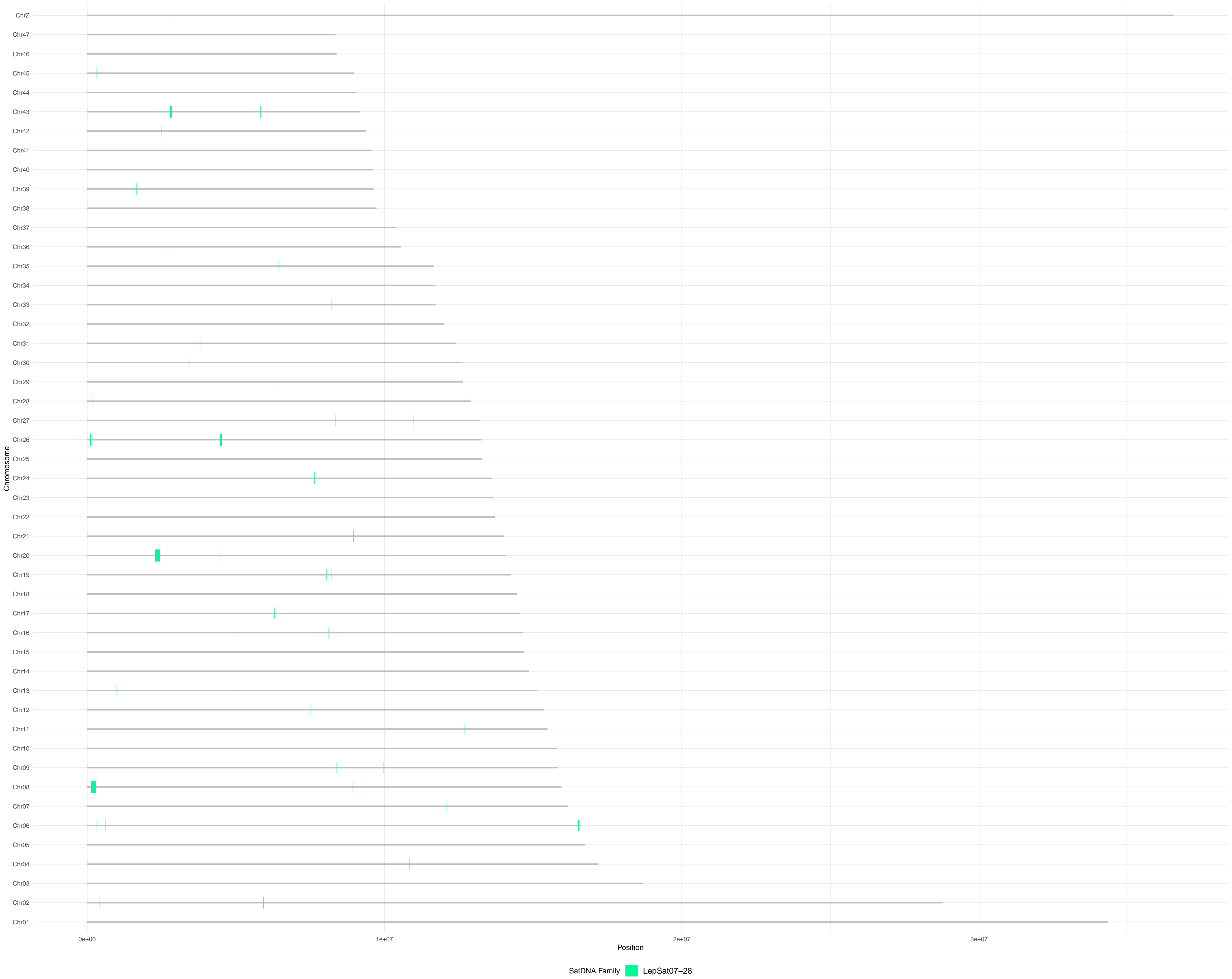

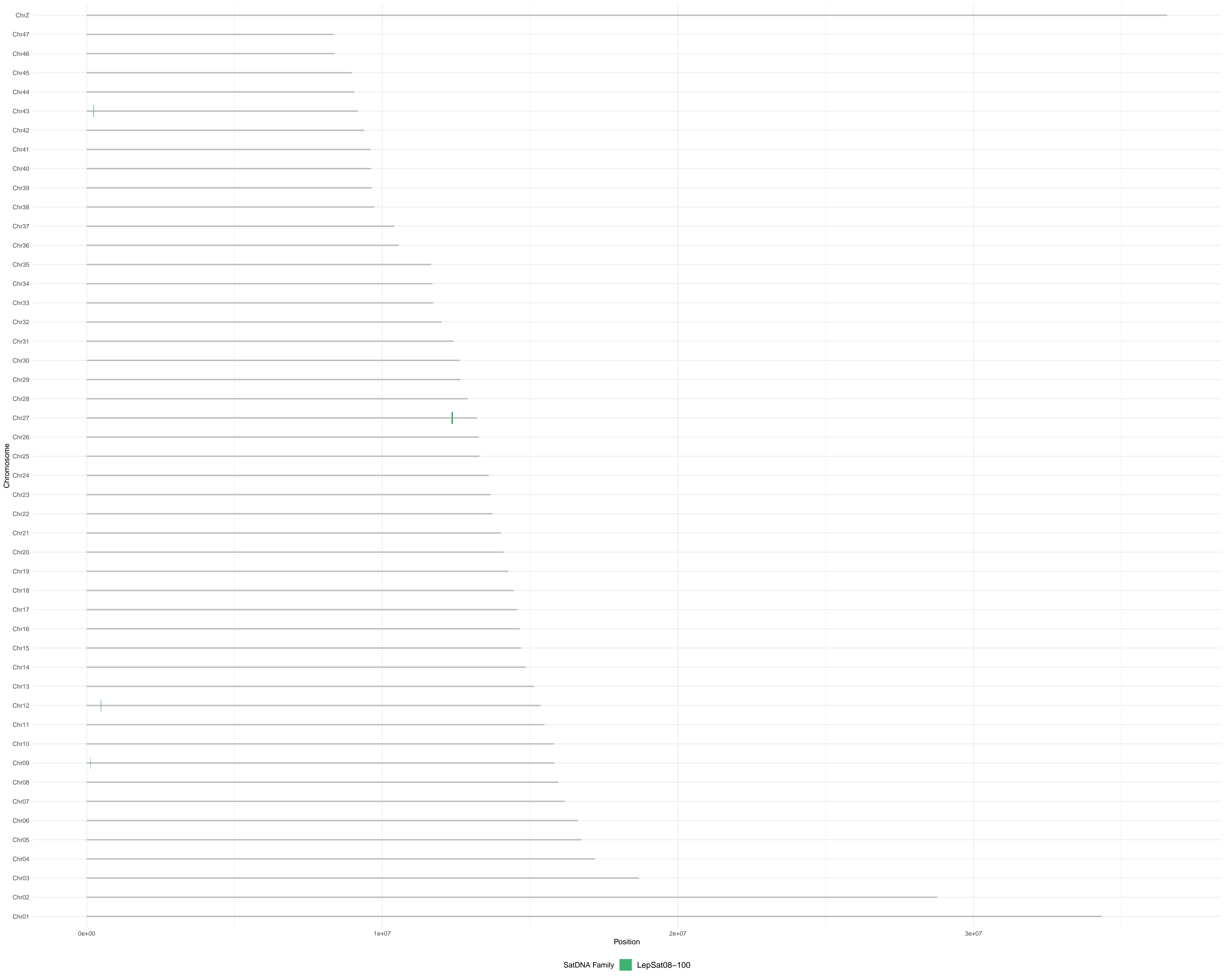

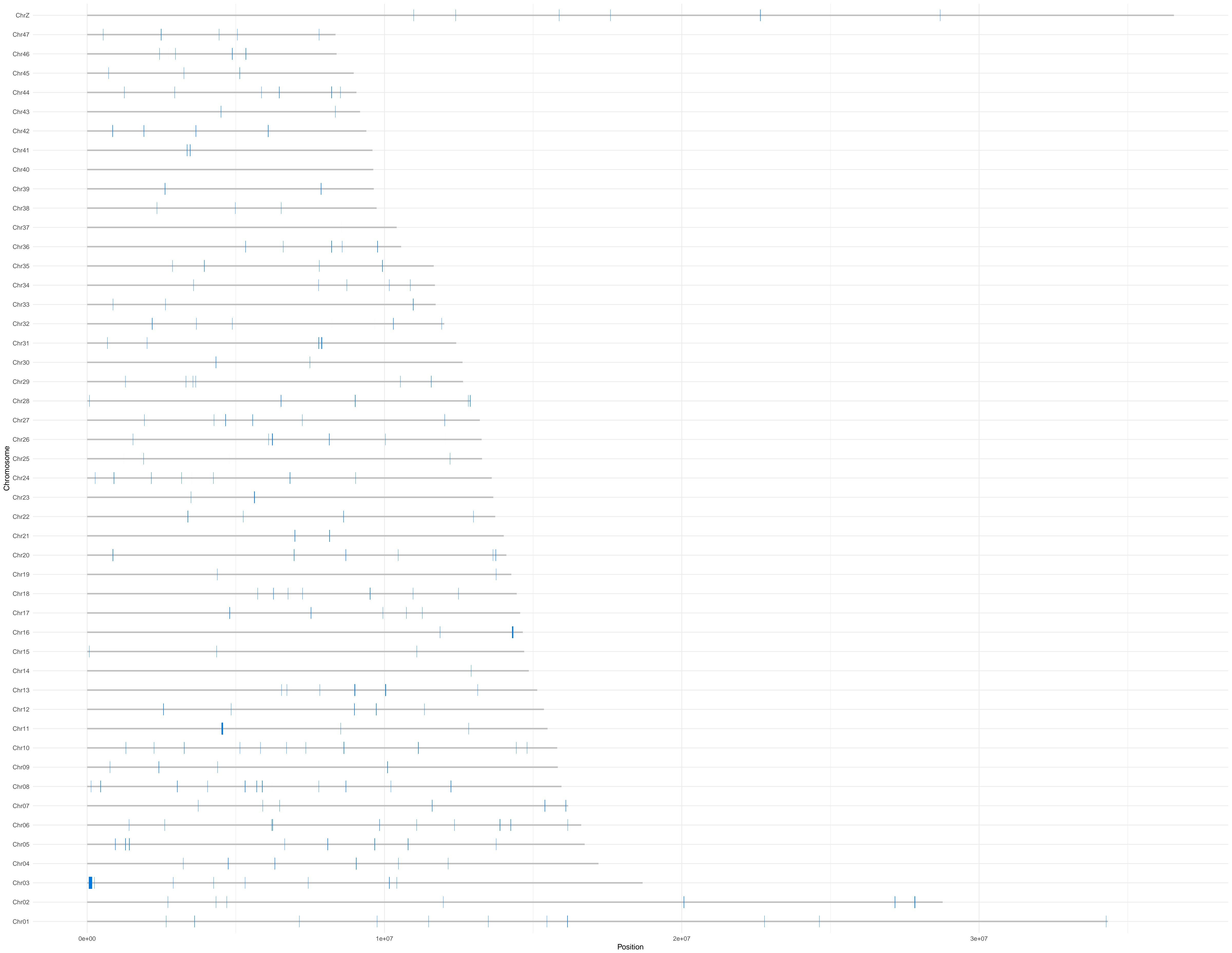

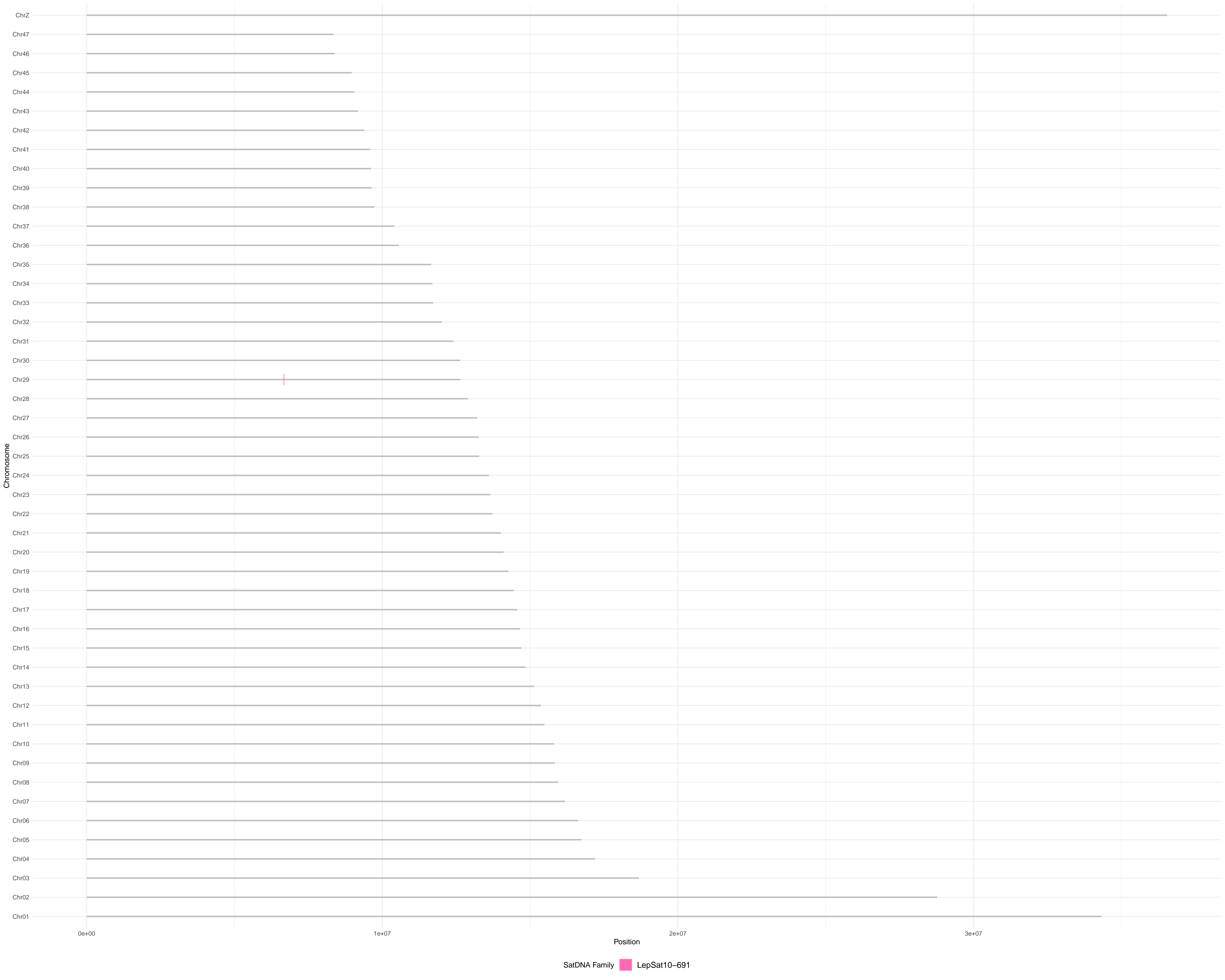

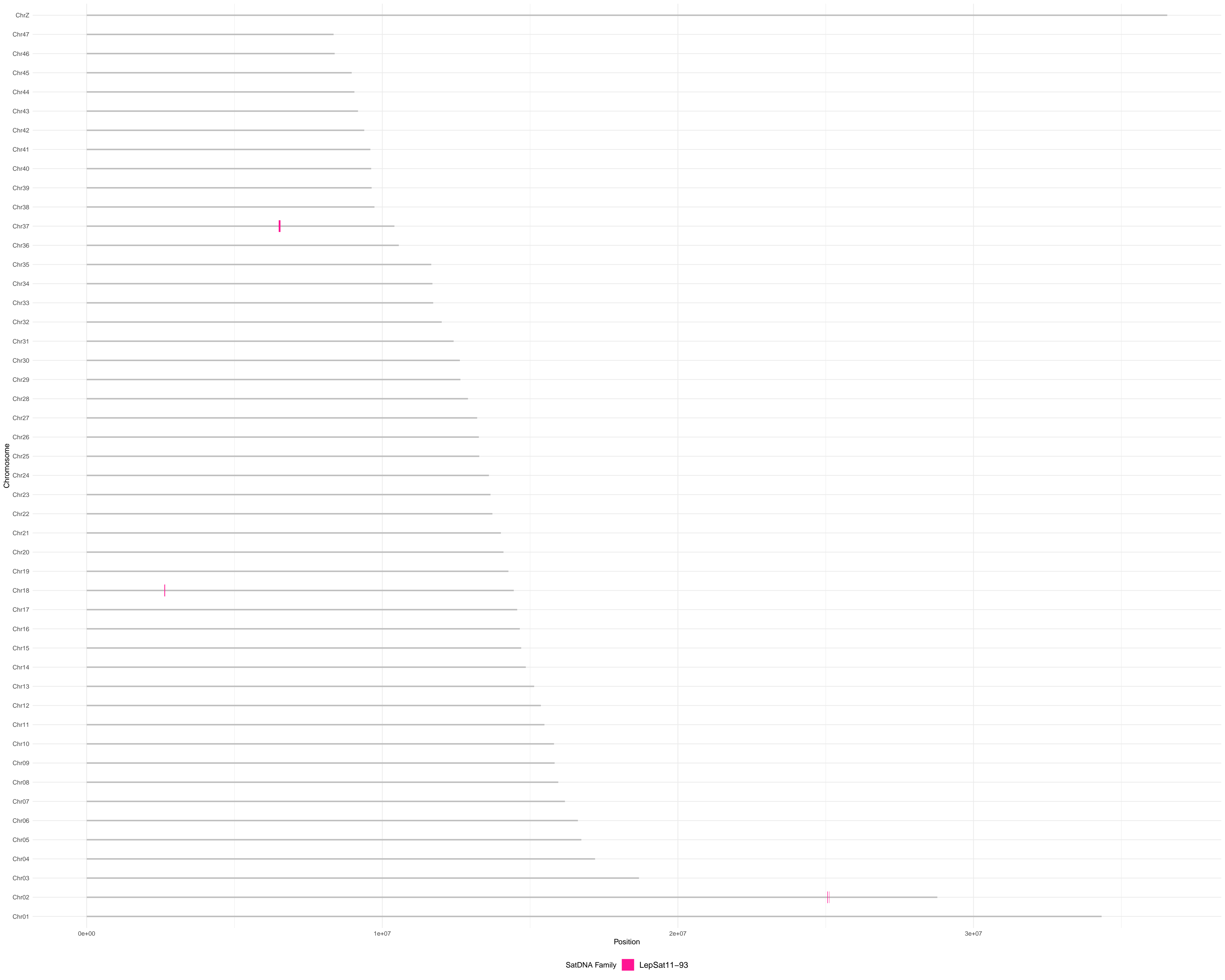

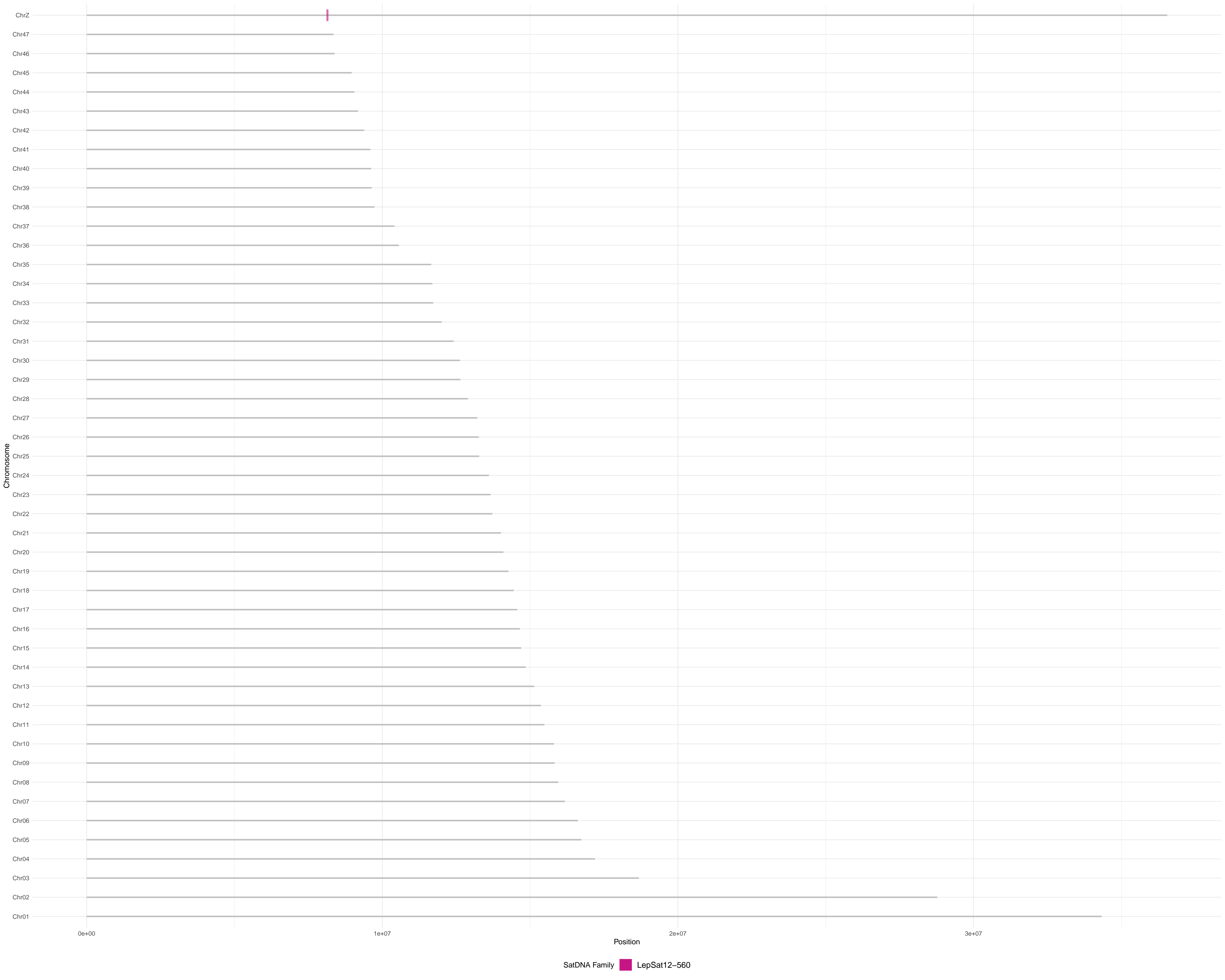

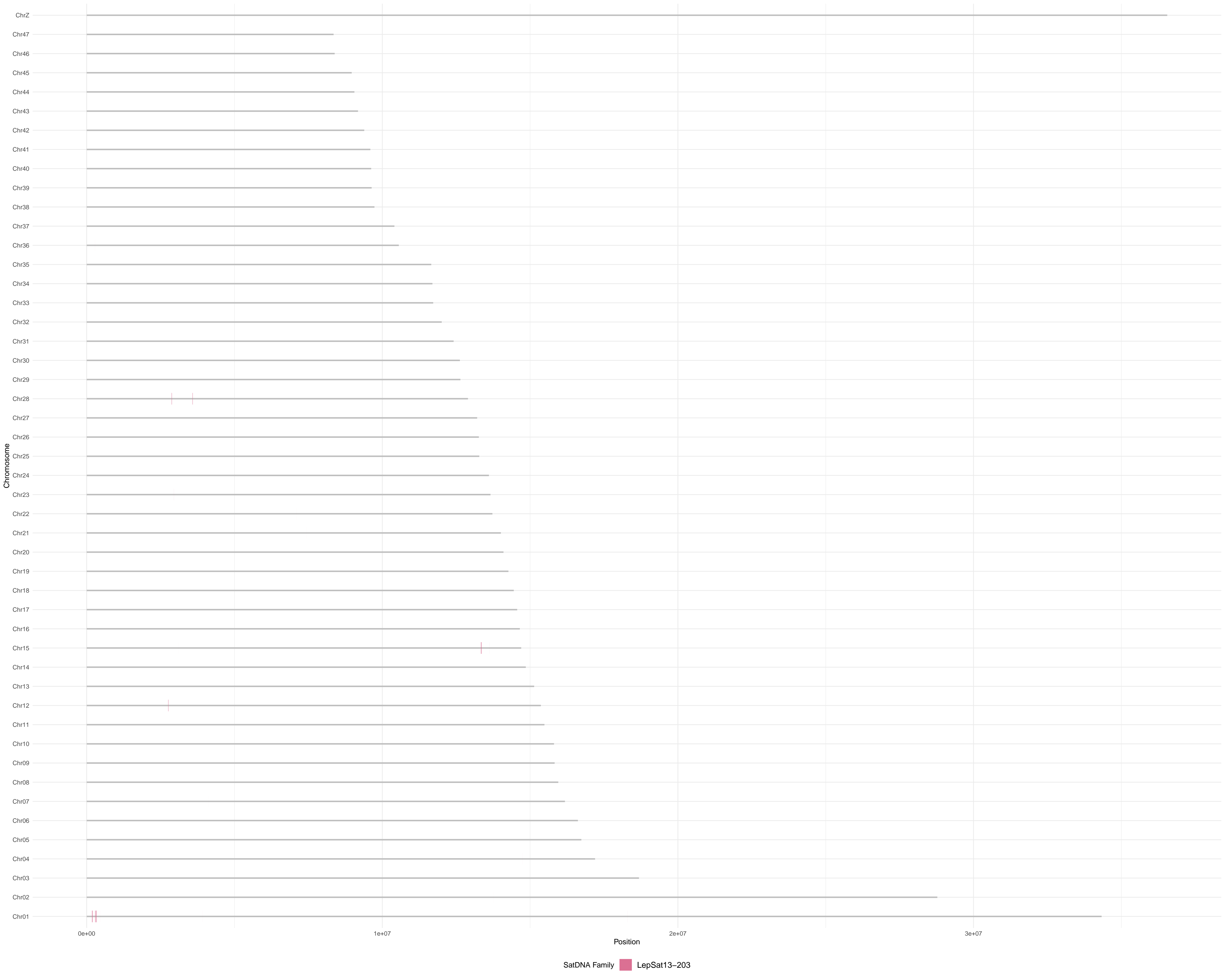

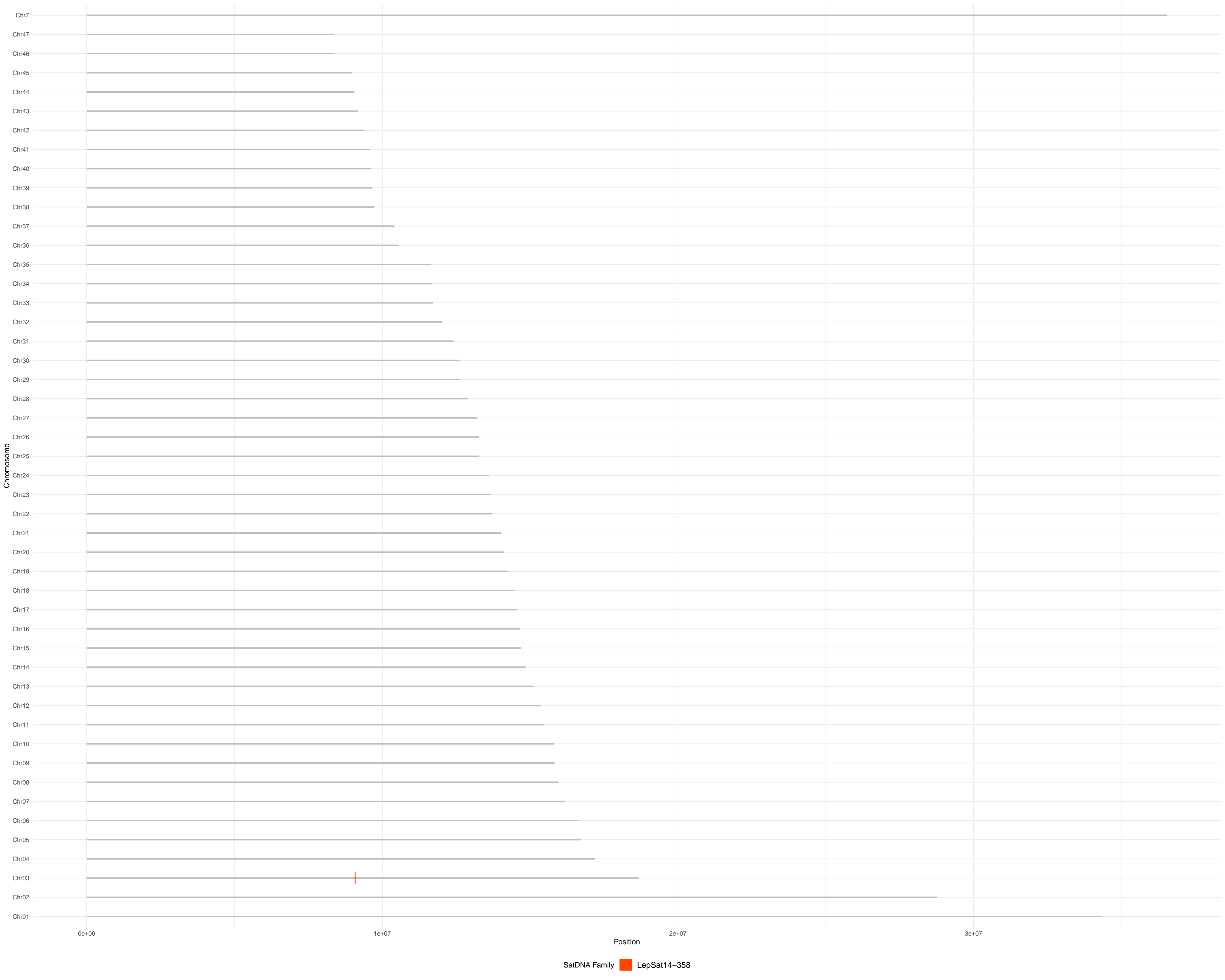

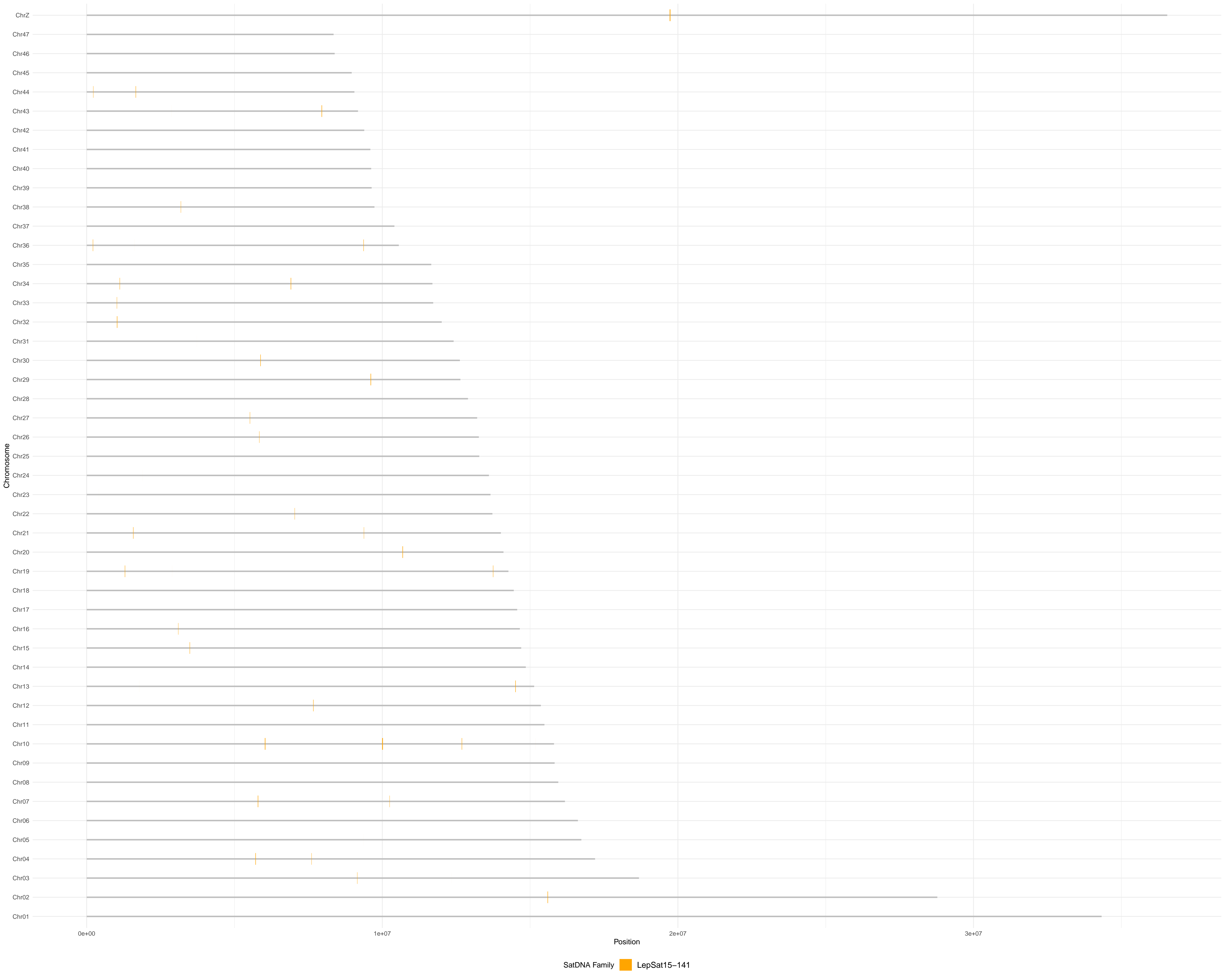

Supplement: dsae030_suppl_Supplementary_Figure_S2 [file dsae030_suppl_supplementary_figure_s2.pdf]
