# Supplementary material for: The burst of satellite DNA in Leptidea wood white butterflies and their putative role in karyotype evolution
Source: DNA Res. 2024 Oct 26;31(6):dsae030. doi: 10.1093/dnares/dsae030 (PMC11565590; doi:10.1093/dnares/dsae030)
Supplement: dsae030_suppl_Supplementary_Table_S1 [file dsae030_suppl_supplementary_table_s1.docx]

**Supplementary Table 1.** List of *Leptidea* genome sequences and their characteristics used for the analysis of satDNAs.

| **SRA number** | **Species (population)** | **Sequencing method** | **Read length** | **Giga bases** | **Data amount used (Gb)** |
| --- | --- | --- | --- | --- | --- |
| ERX2099072 | *L. juvernica* (Ireland) | Illumina HiSeq 2500 | 125 × 2 (pair) | 10.80 | 1.00 |
| ERX2099073 | *L. juvernica* (Ireland) | Illumina HiSeq 2500 | 125 × 2 (pair) | 10.40 | 1.00 |
| ERX2099074 | *L. juvernica* (Ireland) | Illumina HiSeq 2500 | 125 × 2 (pair) | 10.20 | 1.00 |
| ERX2098946 | *L. juvernica* (Kazakhstan) | Illumina HiSeq 2000 | 100 × 2 (pair) | 4.00 | 0.99 |
| ERX2098947 | *L. juvernica* (Kazakhstan) | Illumina HiSeq 2000 | 100 × 2 (pair) | 3.80 | 0.99 |
| ERX2098948 | *L. juvernica* (Kazakhstan) | Illumina HiSeq 2000 | 100 × 2 (pair) | 3.30 | 0.99 |
| ERX2098970 | *L. reali* (Spain) | Illumina HiSeq 2000 | 100 × 2 (pair) | 2.50 | 0.99 |
| ERX2098971 | *L. reali* (Spain) | Illumina HiSeq 2000 | 100 × 2 (pair) | 3.00 | 0.99 |
| ERX2098972 | *L. reali* (Spain) | Illumina HiSeq 2000 | 100 × 2 (pair) | 3.60 | 0.99 |
| ERX2099075 | *L. sinapsis* (Sweden) | Illumina HiSeq 2500 | 125 × 2 (pair) | 10.00 | 1.00 |
| ERX2099076 | *L. sinapsis* (Sweden) | Illumina HiSeq 2500 | 125 × 2 (pair) | 10.20 | 1.00 |
| ERX2099077 | *L. sinapsis* (Sweden) | Illumina HiSeq 2500 | 125 × 2 (pair) | 9.50 | 1.00 |
| ERX2099046 | *L. sinapsis* (Spain) | Illumina HiSeq 2000 | 100 × 2 (pair) | 3.20 | 0.99 |
| ERX2099047 | *L. sinapsis* (Spain) | Illumina HiSeq 2000 | 100 × 2 (pair) | 3.40 | 0.99 |
| ERX2099048 | *L. sinapsis* (Spain) | Illumina HiSeq 2000 | 100 × 2 (pair) | 3.60 | 0.99 |
| ERX11508160 | *L. amurensis* (Mongolia) | Illumina NovaSeq 6000 | 151 × 2 (pair) | 103.20 | 0.91 |
| ERX11508154 | *L. morsei* (China) | Illumina NovaSeq 6000 | 151 × 2 (pair) | 109.70 | 0.91 |
