# Supplementary material for: The burst of satellite DNA in Leptidea wood white butterflies and their putative role in karyotype evolution
Source: DNA Res. 2024 Oct 26;31(6):dsae030. doi: 10.1093/dnares/dsae030 (PMC11565590; doi:10.1093/dnares/dsae030)
Supplement: dsae030_suppl_Supplementary_Table_S2 [file dsae030_suppl_supplementary_table_s2.docx]

**Supplementary Table 2.** Statistical analysis for the satDNAs in *Leptidea* species. Pairwise comparisons of the independent abundance of the 17 satDNAs among the five species were performed using the Wilcoxon matched-pairs test.

|  |  | Statistic | *P* |
| --- | --- | --- | --- |
| *L. juvernica* | *L. reali* | 65.0^a^ | 0.045 |
|  | *L. sinapis* | 82.5^b^ | 0.211 |
|  | *L. amurensis* | 84.5^b^ | 0.173 |
|  | *L. morsei* | 67.5 | 0.687 |
| *L. reali* | *L. sinapis* | 37.5^b^ | 0.211 |
|  | *L. amurensis* | 60.0^d^ | 0.660 |
|  | *L. morsei* | 26.0^e^ | 0.032 |
| *L. sinapis* | *L. amurensis* | 79.0^e^ | 0.587 |
|  | *L. morsei* | 40.0 | 0.089 |
| *L. morsei* | *L. amurensis* | 81.0^d^ | 0.079 |

^a^ 5 pair(s) of values were tied

^b^ 2 pair(s) of values were tied

^d^ 3 pair(s) of values were tied

^e^ 1 pair(s) of values were tied
