# Supplementary material for: The burst of satellite DNA in Leptidea wood white butterflies and their putative role in karyotype evolution
Source: DNA Res. 2024 Oct 26;31(6):dsae030. doi: 10.1093/dnares/dsae030 (PMC11565590; doi:10.1093/dnares/dsae030)
Supplement: dsae030_suppl_Supplementary_Table_S3 [file dsae030_suppl_supplementary_table_s3.docx]

**Supplementary Table 3.** Statistical analysis for the satDNAs in *Leptidea* species. Pairwise comparisons of the independent abundance of the 17 satDNAs between populations of Western Palaearctic species were performed using the Wilcoxon matched-pairs test.

|  |  | Statistic | *P* |
| --- | --- | --- | --- |
| *L. juvernica* (Ireland) | *L. juvernica* (Kazakhstan) | 37.0^a^ | 0.906 |
|  | *L. reali* (Spain) | 96.0^b^ | 0.007 |
|  | *L. sinapis* (Sweden) | 87.0^d^ | 0.132 |
|  | *L. sinapis* (Spain) | 101.0^d^ | 0.021 |
| *L. juvernica* (Kazakhstan) | *L. reali* (Spain) | 70.0^e^ | 0.093 |
|  | *L. sinapis* (Sweden) | 72.5^d^ | 0.495 |
|  | *L. sinapis* (Spain) | 86.0^f^ | 0.365 |
| *L. reali* (Spain) | *L. sinapis* (Sweden) | 43.0^d^ | 0.348 |
|  | *L. sinapis* (Spain) | 45.5^d^ | 0.426 |
| *L. sinapis* (Spain) | *L. sinapis* (Sweden) | 40.5^e^ | 0.753 |

^a^ 5 pair(s) of values were tied

^b^ 3 pair(s) of values were tied

^d^ 2 pair(s) of values were tied

^e^ 4 pair(s) of values were tied

^f^ 1 pair(s) of values were tied
