# Supplementary material for: The burst of satellite DNA in Leptidea wood white butterflies and their putative role in karyotype evolution
Source: DNA Res. 2024 Oct 26;31(6):dsae030. doi: 10.1093/dnares/dsae030 (PMC11565590; doi:10.1093/dnares/dsae030)
Supplement: dsae030_suppl_Supplementary_Table_S4 [file dsae030_suppl_supplementary_table_s4.docx]

**Supplementary Table 4.** Statistical analysis for the satDNAs in *Leptidea* species. Pairwise comparisons of LepSat01-100 abundance between populations of Western Palaearctic species were performed using Student’s *t*-test.

|  |  | Statistic | df | *P* |
| --- | --- | --- | --- | --- |
| *L. juvernica* (Ireland) | *L. juvernica* (Kazakhstan) | -3.67 | 2.00 | 0.067 |
|  | *L. reali* (Spain) | 8.25 | 2.00 | 0.014 |
|  | *L. sinapis* (Sweden) | 16.94 | 2.00 | 0.003 |
|  | *L. sinapis* (Spain) | 17.46 | 2.00 | 0.003 |
| *L. juvernica* (Kazakhstan) | *L. reali* (Spain) | 5.29 | 2.00 | 0.034 |
|  | *L. sinapis* (Sweden) | 7.79 | 2.00 | 0.016 |
|  | *L. sinapis* (Spain) | 9.07 | 2.00 | 0.012 |
| *L. reali* (Spain) | *L. sinapis* (Sweden) | 7.08 | 2.00 | 0.019 |
|  | *L. sinapis* (Spain) | 10.89 | 2.00 | 0.008 |
| *L. sinapis* (Spain) | *L. sinapis* (Sweden) | -14.55 | 2.00 | 0.005 |
