# Supplementary material for: The burst of satellite DNA in Leptidea wood white butterflies and their putative role in karyotype evolution
Source: DNA Res. 2024 Oct 26;31(6):dsae030. doi: 10.1093/dnares/dsae030 (PMC11565590; doi:10.1093/dnares/dsae030)
Supplement: dsae030_suppl_Supplementary_Table_S5 [file dsae030_suppl_supplementary_table_s5.docx]

**Supplementary Table 5.** Statistical analysis for the satDNAs in *Leptidea* species. Pairwise comparisons of LepSat03-167 abundance between populations of Western Palaearctic species were performed using Student’s *t*-test.

|  |  | Statistic | df | *P* |
| --- | --- | --- | --- | --- |
| *L. juvernica* (Ireland) | *L. juvernica* (Kazakhstan) | 7.7798 | 2.00 | 0.016 |
|  | *L. reali* (Spain) | 1.2898 | 2.00 | 0.326 |
|  | *L. sinapis* (Sweden) | -12.9996 | 2.00 | 0.006 |
|  | *L. sinapis* (Spain) | 0.0996 | 2.00 | 0.930 |
| *L. juvernica* (Kazakhstan) | *L. reali* (Spain) | -5.1590 | 2.00 | 0.036 |
|  | *L. sinapis* (Sweden) | -19.4523 | 2.00 | 0.003 |
|  | *L. sinapis* (Spain) | -3.2858 | 2.00 | 0.081 |
| *L. reali* (Spain) | *L. sinapis* (Sweden) | -7.4696 | 2.00 | 0.017 |
|  | *L. sinapis* (Spain) | -0.5510 | 2.00 | 0.637 |
| *L. sinapis* (Spain) | *L. sinapis* (Sweden) | -3.9173 | 2.00 | 0.059 |
