# Supplementary material for: The burst of satellite DNA in Leptidea wood white butterflies and their putative role in karyotype evolution
Source: DNA Res. 2024 Oct 26;31(6):dsae030. doi: 10.1093/dnares/dsae030 (PMC11565590; doi:10.1093/dnares/dsae030)
Supplement: dsae030_suppl_Supplementary_Material [file dsae030_suppl_supplementary_material.docx]

**Supplementary Material**

**Supplementary Table 1.** List of *Leptidea* genome sequences and their characteristics used for the analysis of satDNAs.

**Supplementary Table 2.** Statistical analysis for the satDNAs in *Leptidea* species. Pairwise comparisons of the independent abundance of the 17 satDNAs among the five species were performed using the Wilcoxon matched-pairs test.

**Supplementary Table 3.** Statistical analysis for the satDNAs in *Leptidea* species. Pairwise comparisons of the independent abundance of the 17 satDNAs between populations of Western Palaearctic species were performed using the Wilcoxon matched-pairs test.

**Supplementary Table 4.** Statistical analysis for the satDNAs in *Leptidea* species. Pairwise comparisons of LepSat01-100 abundance between populations of Western Palaearctic species were performed using Student’s *t*-test.

**Supplementary Table 5.** Statistical analysis for the satDNAs in *Leptidea* species. Pairwise comparisons of LepSat03-167 abundance between populations of Western Palaearctic species were performed using Student’s *t*-test.

**Supplementary Figure 1.** Electrophoresis on agarose gel 1.5% for the PCR products of LepSat01-100 and LepSat03-167 in the three Western Palaearctic *Leptidea* species. The first lane of each image represents the marker (M), which indicates the size of the DNA bands in base pairs (bp). Note the ladder pattern of amplification for both satDNAs in the three species, including monomers, dimers, trimers and multimers, which is a characteristic pattern for satDNAs.

**Supplementary Figure 2.** CHRISMAPP output for all satDNAs separately on the assembled chromosomes of *Leptidea sinapis*. ChrZ, Chr02 and Chr03 correspond to Z_1_, Z_2_ and Z_3_, respectively.
